# From Clustered to Sporadic: Structural Shifts in the Spatiotemporal Dynamics of HPAI Following the 2017 Policy Reinforcement in South Korea (2003–2025)

**DOI:** 10.1155/tbed/5747471

**Published:** 2026-07-07

**Authors:** Sung Dae Park, Ji Hyun Son, Dae Sung Yoo

**Affiliations:** ^1^ Animal Health Policy Division, Ministry of Agriculture, Food and Rural Affairs, Sejong, Republic of Korea, omafra.gov.on.ca; ^2^ Graduate School of Public Administration, Korea University, Sejong, Republic of Korea, korea.ac.kr; ^3^ FMD and Large Animal Health Control Division, Ministry of Agriculture, Food and Rural Affairs, Sejong, Republic of Korea, omafra.gov.on.ca; ^4^ Department of Veterinary Epidemiology, College of Veterinary Medicine, Chonnnam National University, Gwangju, Republic of Korea

**Keywords:** control policy evaluation, highly pathogenic avian influenza (HPAI), space–time *k*-function, space–time permutation scan statistics (STPSS), spatiotemporal analysis

## Abstract

Highly pathogenic avian influenza (HPAI) is a devastating viral disease causing substantial economic losses in the poultry industry and posing potential zoonotic risks. Located along the East Asian–Australasian Flyway (EAAF), South Korea has experienced recurrent outbreaks of HPAI since 2003. Following the severe 2016–2017 epidemic, the government implemented strengthened control measures, including restrictions on duck farming and organizational restructuring. This study quantitatively evaluated structural changes in the spatiotemporal patterns and transmission dynamics of HPAI before and after the 2017 policy reinforcement, utilizing a complete dataset covering 12 epidemic waves between 2003 and 2025. Our analysis suggests a distinct shift in HPAI occurrence patterns from large‐scale, clustered epidemics to more sporadic occurrences in the post‐2017 period. Pre‐2017 epidemics, particularly the 5th and 6th waves, exhibited intense spatiotemporal clustering and high transmission potential. Conversely, post‐2017 epidemics showed a significant reduction in outbreak density and the disappearance of large‐scale clusters. Notably, the 12th wave displayed a more circular diffusion pattern with outbreaks confined to specific regions, suggesting relatively more geographically contained spread. However, despite the overall reduction in scale, high spatiotemporal interaction intensity was intermittently observed, such as in the 11th wave, indicating that residual risks of explosive local transmission persist even during smaller epidemics. These findings suggest that the post‐2017 pattern was temporally consistent with strengthened control policies aimed at reducing mechanical connectivity between farms, although this ecological analysis cannot separate policy effects from other time‐varying epidemiological and surveillance‐related factors. Nevertheless, a decline in case numbers does not necessarily imply the elimination of local transmission risk, highlighting the need to advance precise and risk‐based surveillance and response strategies to effectively manage residual risks.

## 1. Introduction

Highly pathogenic avian influenza (HPAI) is a zoonotic disease that causes significant losses in poultry productivity and international trade and also poses public health risks through human infection [1]. Since 2020, the global expansion of H5 subtypes, predominantly clade 2.3.4.4b, detected not only in birds but increasingly in various mammals, has reemphasized the need for risk management at the human–animal interface [1–4]. Located at the center of the East Asian–Australasian Flyway (EAAF), South Korea is structurally vulnerable to the recurrent introduction of HPAI viruses due to migratory bird movements and interconnected wetland ecosystems. Consequently, the country has experienced repeated epidemics associated with seasonal migration since 2003, resulting in substantial socioeconomic costs, including large‐scale culling, movement restrictions, and consumption contraction [5].

The spread of HPAI is generally described by a two‐stage mechanism: (1) primary introduction from wild birds [6] and (2) secondary spread through mechanical transmission between farms [7]. In the introduction phase, viral detection in wild birds and farm outbreaks has been reported to show seasonal correlations [8, 9]. In the expansion phase, vectors such as humans, vehicles, and equipment create connectivity between farms, facilitating local and regional transmission [10]. Domestic ducks are recognized as a risk factor for “silent transmission” because they can shed the virus and transmit it among individuals while exhibiting mild or unclear clinical symptoms, thereby complicating early detection and containment [11, 12].

Following the severe winter epidemic of 2016–2017, South Korea significantly strengthened its disease control system. The government reorganized its control structure by establishing the Animal Health Policy Bureau within the Ministry of Agriculture, Food and Rural Affairs in August 2017 [13]. Furthermore, high‐intensity measures were implemented in November 2017, such as the “Duck Farming Restriction” policy, which restricts duck farming in high‐risk areas during the winter season (November to February) in exchange for compensation [14]. However, there is a lack of accumulated quantitative evidence assessing how these policy reinforcements have fundamentally altered long‐term spatiotemporal outbreak patterns, such as shifts from large‐scale clustered outbreaks to more sporadic cases, beyond analyses focused on single epidemics or short time series.

Policy evaluation is a critical process for ensuring accountability in interventions and efficiency in resource allocation. Given the direct and indirect costs associated with mass culling and movement restrictions, evidence‐based evaluation that quantifies the epidemiological effects of interventions is essential [15]. Accordingly, this study used complete outbreak data from 2003 to 2025 (12 epidemic waves) to compare and evaluate changes in the spatiotemporal cluster structure of domestic HPAI before and after 2017. The space–time permutation scan statistic (STPSS) is a valuable method for detecting clusters using case‐only data, particularly when the population at risk fluctuates significantly across space and time or is difficult to estimate accurately [16–18]. This study analyzed (1) changes in the size, duration, and spatial distribution of significant clusters by epidemic wave; (2) shifts in spatial concentration and diffusion directionality before and after 2017; (3) interepidemic differences in transmission aggregation (intensity) indices based on the space–time *K*‐function. Through this approach, we aimed to identify structural changes in high‐risk periods and regions from a long‐term perspective and to provide empirical evidence necessary for designing customized and risk‐based control strategies.

## 2. Materials and Methods

### 2.1. Data Collection and Processing

This study analyzed data from 12 epidemic waves of HPAI in poultry farms in South Korea from December 2003 to June 2025. Information on confirmation date, livestock species, serotype, and the address of infected farms was collected from the Korea Animal Health Integrated System and internal records of the Ministry of Agriculture, Food and Rural Affairs. Address data were geocoded to obtain coordinates and subsequently transformed into planar coordinates (*x*, *y*) using the Korean projected coordinate system (EPSG: 5179; units in meters) in QGIS to establish a spatial database. Administrative boundary data (shapefiles) used for spatial analysis and visualization were obtained from the Statistical Geographic Information Service (SGIS) provided by the Ministry of Data and Statistics [19]. All distance‐based spatial analyses in this study, including radius settings for the STPSS and distance criteria for kernel density estimation (KDE), standard deviational ellipse (SDE), and spatiotemporal *K* function, were performed using the EPSG: 5179 (Korea 2000/Unified CS) coordinate system. To ensure precision in temporal analysis, all data were aggregated on a daily basis.

### 2.2. Spatiotemporal Cluster Detection

To detect spatiotemporal clusters of HPAI outbreaks, STPSS was employed using SaTScan (v10.3.2) software for the analysis [16]. The STPSS model is a valid method for detecting clusters resulting from spatiotemporal interaction, adjusting for purely spatial and purely temporal variations using only case data without requiring population data [16, 20]. This model has the advantage of selectively identifying actual disease outbreaks resulting from spatiotemporal interaction by adjusting for simple spatial clustering effects, such as high‐density farming areas, and temporal clustering effects, such as seasonality [16]. Furthermore, it is known to produce relatively robust results even when data are incomplete or contain missing values [21]. The STPSS method begins by defining numerous theoretical cylindrical scan windows that cover the entire study area and period [22, 23]. The circular base of each cylinder represents the candidate area for a spatial cluster and the height represents the candidate duration for a temporal cluster. During the analysis, the location and size (radius and height) of these cylinders are continuously varied to explore all possible spatiotemporal combinations [24]. For each cylindrical window *Z*, the null hypothesis (*H*_0_) assumes independence between the spatial and temporal distributions of cases, whereas the alternative hypothesis (*H*_1_) assumes that the risk of disease occurrence is higher inside the cylinder than outside. The STPSS model calculates test statistics based on permutation, which assumes that the timing of an outbreak at a specific farm is independent of the locations of other farms. The most likely cluster is identified as the cylinder *Z* that maximizes the log likelihood ratio (LLR). The LLR is expressed by the following equation [16]:

LLR=logcZEcZcZC−cZC−EcZC−cZ.

if cZ>EcZ.

Here, *C* denotes the total number of cases in the dataset, *c*_*Z*_ is the observed number of cases within cylinder *Z*, and *E*[*c*_*Z*_] is the expected number of cases within *Z* under *H*_0_. Under the space–time permutation model, *E*[*c*_*Z*_] is computed by SaTScan based on the observed spatial and temporal marginal totals, rather than on an external population at risk. The LLR quantifies how anomalously high the observed number of cases is compared with the expected value. The statistical significance of the calculated maximum LLR value is evaluated using Monte Carlo hypothesis testing [16]. This process involves generating numerous synthetic datasets that follow the null hypothesis by randomly reordering the timestamps of all outbreak cases and repeatedly calculating the maximum LLR for each dataset. The *p*‐value is calculated by comparing the maximum LLR from the observed data with the distribution of LLR values obtained from the random datasets. In this study, the *p*‐value was calculated using 9999 simulations, and clusters were considered statistically significant if the *p*‐value was less than 0.05. To maintain consistency in analysis and compare results across 12 epidemic waves, identical parameters were applied to all analyses [25]. The maximum size of the spatial scan window was limited to 20% of the total number of cases to focus on detecting local hot spots amenable to policy intervention rather than large‐scale clusters at the national level [26]. The maximum search radius was set to 40 km based on the average movement range of domestic livestock vehicles identified in previous studies and the criteria for establishing disease control zones [27, 28]. This setting was intended to standardize the detection of local and regional clusters under a comparable framework across epidemic waves. However, because the present analysis is based on distance‐defined spatial windows, it is better suited to detecting localized spatiotemporal clustering than reconstructing long‐distance transmission mediated by transportation or production networks. Accordingly, noncontiguous mechanical transmission chains may be underdetected or misclassified in the present framework. Notably, a study analyzing the spatiotemporal *K* function for the H5N8 epidemic in South Korea from 2014 to 2016 confirmed that statistically significant spatiotemporal interaction was maintained up to a 40 km range, supporting the epidemiological validity of this radius setting [26]. The maximum size of the temporal window was limited to 30% of the duration of each wave to detect both short‐term explosive outbreaks and long‐term epidemics [25]. The minimum time unit was set to 7 days, considering the average time required to complete initial control measures, such as culling and disinfection, following HPAI occurrence (Table 1).

**Table 1:** Parameter settings for the space–time permutation scan statistic (STPSS) by epidemic wave.

Wave	Period (days)	Cases	Max. temporal (30%)	Min. temporal (days)	Max. spatial (%)	Radius (km)
1st	2003/12/10–2004/03/20 (102)	19	31	7	20	40
2nd	2006/11/22–2007/03/20 (119)	13	36	7	20	40
3rd	2008/04/01–2008/05/24 (54)	98	16	7	20	40
4th	2010/12/29–2011/05/21 (144)	91	43	7	20	40
5th	2014/01/16–2016/04/05 (811)	393	243	7	20	40
6th	2016/11/16–2017/06/19 (216)	419	65	7	20	40
7th	2017/11/17–2018/03/17 (121)	22	36	7	20	40
8th	2020/11/26–2021/04/06 (132)	109	40	7	20	40
9th	2021/11/08–2022/04/07 (151)	47	45	7	20	40
10th	2022/10/17–2023/04/14 (180)	75	54	7	20	40
11th	2023/12/03–2024/05/22 (172)	32	52	7	20	40
12th	2024/10/29–2025/06/27 (242)	49	73	7	20	40

### 2.3. Macrospatial Analysis: KDE ad SDE

To identify the overall intensity and directionality of HPAI outbreaks, KDE and SDE analyses were performed using QGIS. KDE generated a density surface based on the spatial distribution of outbreak points for each epidemic wave, and the maximum value at the point of highest density was extracted to quantify the concentration of outbreaks by wave. A Gaussian kernel was applied within the projected coordinate system (EPSG: 5179). To ensure quantitative comparability across all 12 waves, a primary bandwidth of 10 km was used, corresponding to the radius of the surveillance zone defined in the national HPAI control policy, together with a raster cell size of 100 m. Because the poultry industry in South Korea is spatially heterogeneous, we additionally conducted a sensitivity analysis using Gaussian kernel bandwidths of 5, 10, and 15 km to assess whether the wave‐level contrast in outbreak density was robust to the smoothing parameter [29, 30].

SDE was used to derive the centrality, dispersion, and directionality of the outbreak distribution in the form of an ellipse. Each outbreak point was assigned with equal weight, and the SDE calculated the mean center and covariance‐based major and minor axes, producing a one standard deviation (1‐SD) ellipse. As a quantitative indicator of diffusion directionality, eccentricity $e=\sqrt{1-{\left(b/a\right)}^{2}}$ (where *a* is the semimajor axis and *b* is the semiminor axis) was calculated from the lengths of the axes. An eccentricity value closer to 1 was interpreted as linear diffusion, whereas a value closer to 0 was interpreted as circular diffusion [30, 31].

### 2.4. Spatiotemporal Transmission Dynamics: STK Function

To quantitatively evaluate short‐range spatiotemporal interaction beyond simple case counts, a space–time *K*‐function (*K*) analysis was conducted. The space–time *K*‐function *K*(*s*, *t*) represents the expected number of events, or expected cumulative frequency, occurring within a distance *s* and time *t* of an arbitrary event. The magnitude of spatiotemporal interaction is expressed as *D*(*s*, *t*) = *K*(*s*, *t*) − *K*_*s*_(*s*)*K*_*t*_(*t*). A value of *D*(*s*, *t*) > 0 indicates the presence of “excess” clustering or interaction that cannot be explained solely by the independent distributions of space and time [32, 33].

In this study, *D*(*s*, *t*) values within a 3 km radius and 6 days were extracted to compare and analyze initial transmission clustering and transmission intensity for each epidemic. For brevity, *D*(3 km, 6 days) is hereafter referred to as the “STK value” and was used as a wave‐level summary metric of early local spatiotemporal interaction. The 3 km distance corresponds to the policy unit for domestic control zones, and the 6‐day period was defined as an “early interaction window,” considering the incubation period and potential delays in reporting or initial response. This setting was intended to capture short‐range spatiotemporal clustering under a comparable standard across waves. Importantly, elevated STK values do not by themselves distinguish true secondary farm‐to‐farm transmission from near‐simultaneous outbreaks arising from common‐source exposure. The significance of *D*(*s*, *t*) was evaluated using a null distribution generated by Monte Carlo simulations under the assumption of random rearrangement of occurrence times or spatiotemporal independence, and standard correction methods were applied for edge effects [33, 34]. Through this approach, the intensity of local spatiotemporal interaction, rather than the direct transmission sequence, was quantitatively compared and evaluated. This method has been proven effective for analyzing the spread patterns of poultry diseases and has been successfully applied to quantify the range and intensity of spatiotemporal interactions in HPAI H5N1 epidemics in Vietnam [34].

### 2.5. Geographic Information System (GIS) Visualization

To effectively interpret and communicate the results of statistically significant spatiotemporal clusters derived from SaTScan, multilayered visualization was performed using the GIS software QGIS (v3.44). The primary objective of GIS visualization is to transform complex spatiotemporal data into an intuitively understandable format and to explore the geographical characteristics of HPAI occurrence patterns in depth [30].

The visualization process was conducted as follows: First, the locations of individual infected farms for each of the 12 epidemic waves were plotted as point data to identify the overall spatial distribution. Second, significant clusters identified by SaTScan analysis were visualized as circles, with the center of each circle representing the geographic center of the cluster and the radius indicating its spatial extent. This enabled clear confirmation of the location and size of clusters as well as their spatial relationships with other clusters and infected farms.

Furthermore, the principles of choropleth mapping were applied to visually distinguish the temporal characteristics and risk levels of clusters. The color transparency of each cluster circle was set to be proportional to its relative risk (RR) value, such that clusters with higher risk were displayed using more opaque and darker colors. Additionally, to represent the temporal sequence of cluster occurrences within each epidemic, a sequential color scheme was applied, ranging from light colors for early outbreaks to dark blue for later outbreaks. This enabled the dynamic visualization of the spatiotemporal progression of the epidemic. These maps played a key role in visually comparing the unique diffusion pattern of each wave and in clearly revealing changes in patterns before and after the strengthening of control policies.

In addition to cluster detection, the results of the macrospatial analysis were also visualized to depict the overall structural changes. Density surfaces derived using KDE were rendered to highlight high‐density hot spots, and SDEs were overlaid on the map as 1‐SD ellipses. This visualization approach effectively illustrated the spatial centrality and directionality of the spread for each epidemic wave, complementing the local cluster analysis.

## 3. Results

### 3.1. Local Clustering Patterns

The STPSS analysis identified 43 statistically significant high‐risk clusters (*p*  < 0.05) out of a total of 117 potential clusters (Supporting Information 1: Table S1). Distinct differences in cluster formation patterns were observed across epidemic waves (Supporting Information 3: Figure S1).

First, with respect to clustered epidemics, the 5th (H5N8) and 6th (H5N6) waves, which were the largest recorded epidemics, exhibited multiple strong cluster formations, with 17 and 13 clusters being detected, respectively. Notably, during the 5th wave, explosive clustering was detected within a localized area of less than 1 km radius, with a risk 131 times higher than expected. This indicates intense short‐range spatiotemporal interactions consistent with local amplification among nearby farms.

In contrast, no significant clusters were detected in the 7th, 8th, and 9th waves, which occurred after the 2017 policy reinforcement, suggesting a transition toward less sustained wave‐level spatiotemporal clustering. However, in later post‐2017 waves, high short‐range spatiotemporal interaction (STK) was observed in some instances, indicating that the absence of STPSS‐detected clusters does not necessarily preclude explosive local transmission once the virus is introduced into vulnerable and high‐density settings. Overall, these findings are consistent with a shift toward more dispersed occurrence patterns at the wave level, with a residual risk of localized amplification (Table 2).

**Table 2:** Epidemiological characteristics and spatiotemporal cluster patterns of highly pathogenic avian influenza (HPAI) waves.

Wave	Subtype	Epidemiological characteristics
1st	H5N1	(Small‐scale concentrated outbreaks) Although there were only 19 cases a significant cluster (relative risk [RR] = 6.3) formed within a narrow 1.87 km radius in the Eumseong, Chungbuk region during the early phase, indicating a pattern of intensive initial transmission.
2nd	H5N1	(Small‐scale sporadic outbreaks) A very low number of cases (13) occurred, and no statistically significant clusters were detected.
3rd	H5N1	(Nationwide spread via multiple clusters) As the number of cases increased to 98 four significant clusters appeared simultaneously across the country, including in Gyeongsang, Jeolla, and Chungcheong provinces. Some clusters reached an RR of 11.7, suggesting strong intra‐regional transmission.
4th	H5N1	(Spread of high‐risk, small‐scale clusters) A total of 91 cases occurred, with five significant clusters detected. Clusters in Gyeongsang and northern Gyeonggi had narrow radii (less than 5 km) but high RRs (18.2), indicating very high transmissibility.
5th	H5N8	(Unprecedented epidemic and explosive transmission) This massive outbreak involved 393 cases and 17 significant clusters nationwide. Ultra‐small clusters (radius <1 km) exhibited RRs of up to 131, reflecting a complex epidemic pattern of simultaneous explosive local transmission and widespread dissemination.
6th	H5N6/H5N8	(Largest recorded epidemic) The largest wave recorded, with 419 cases and 13 significant clusters. It began with a strong primary cluster (RR = 12.2) in the Chungcheong region and spread nationwide, including the emergence of a high‐risk cluster (RR = 41.9) in Jeju.
7th	H5N6	(Subsidence following a major epidemic) Occurring immediately after the massive 6th wave, this epidemic involved 22 cases and formed no significant clusters. This suggests that strengthened control policies successfully blocked local transmission.
8th	H5N8	(Medium‐scale epidemic without clusters) Despite 109 cases, no statistically significant clusters were detected, suggesting weaker and/or less coherent wave‐level space–time interaction compared with earlier epidemics.
9th	H5N1	(Small‐scale epidemic without clusters) With 47 cases a sporadic pattern with a high likelihood of individual farm infections was observed.
10th	H5N1	(Reemergence of cluster patterns) Significant clusters reappeared after several years of sporadic epidemics. Two broad clusters were identified in the Chungcheong and Jeonnam regions.
11th	H5N1/H5N6	(Reconfirmation of sporadic patterns) Following the reemergence of clusters in the 10th wave, the pattern returned to small‐scale sporadic occurrences with 32 cases.
12th	H5N1	(Reemergence of cluster patterns) A single cluster was formed in the Chungcheong region.

### 3.2. Macrospatial Structural Shifts

The KDE analysis revealed a marked variation in outbreak intensity across epidemic waves (Supporting Information 4: Figure S2). The 5th wave recorded the highest KDE maximum (117.21), indicating an extremely strong spatial concentration within localized areas. In contrast, the 8th wave showed a comparatively low KDE maximum (3.69) despite involving 109 cases. This pattern remained qualitatively consistent across sensitivity analyses using 5, 10, and 15 km bandwidths (Supporting Information 2: Table S2), supporting the interpretation that the relatively low spatial concentration of the 8th wave was not solely a smoothing artifact.

Significant changes in diffusion directionality were confirmed through SDE analysis. Most epidemic waves exhibited strong linearity, with eccentricity values greater than 0.85 along the north–south axis, reflecting the topography of the Korean Peninsula and migratory bird flyways. However, the most recent 12th wave (2024/25) showed an eccentricity of 0.738, the lowest recorded value. This suggests that viral spread was more geographically confined within specific regions. This pattern is temporally consistent with strengthened movement control, but it should not be interpreted as direct evidence of the policy effect alone (Table 3).

**Table 3:** Summary of spatial statistics for HPAI epidemic waves (1st–12th): KDE maximum (10 km bandwidth), SDE eccentricity, and spatiotemporal interaction (STK value).

Wave	Period (days)	Cases	KDE max.	SDE eccentricity	STK value	Characteristic of spread
1st	2003/12/10–2004/03/20 (102)	19	3.35	0.867	9.80 × 10^10^	Early cluster (high interaction)
2nd	2006/11/22–2007/03/20 (119)	13	4.94	0.981	2.40 × 10^10^	Linear sporadic (high linearity)
3rd	2008/04/01–2008/05/24 (54)	98	32.03	0.801	2.59 × 10^11^	Regional outbreak
4th	2010/12/29–2011/05/21 (144)	91	9.58	0.917	1.40 × 10^11^	Strong propagation
5th	2014/01/16–2016/04/05 (811)	393	117.21	0.946	7.30 × 10^11^	Explosive (Max. density and interaction)
6th	2016/11/16–2017/06/19 (216)	419	35.11	0.930	1.57 × 10^11^	Widespread (massive but dispersed)
7th	2017/11/17–2018/03/17 (121)	22	1.85	0.984	1.47 × 10^10^	Controlled (low density, high linearity)
8th	2020/11/26–2021/04/06 (132)	109	3.69	0.891	5.41 × 10^9^	Sporadic (lowest interaction)
9th	2021/11/08–2022/04/07 (151)	47	3.84	0.922	3.52 × 10^10^	Sporadic
10th	2022/10/17–2023/04/14 (180)	75	4.88	0.858	8.39 × 10^10^	Reemerging cluster
11th	2023/12/03–2024/05/22 (172)	32	6.46	0.858	4.67 × 10^11^	High local interaction (small scale)
12th	2024/10/29–2025/06/27 (242)	49	4.87	0.738	1.42 × 10^10^	Geographically confinement pattern

*Note*: KDE max indicates the highest spatial concentration of outbreaks and is based on the primary 10 km bandwidth specification. The robustness of KDE–based comparisons was evaluated through sensitivity analyses using 5 and 15 km bandwidths (Supporting Information 1: Table S2) SDE eccentricity ranges from 0 (circular/local diffusion) to 1 (linear/elongated diffusion). STK value denotes the strength of spatiotemporal interaction summarized as *D* (3 km, 6 days), where *D*(*s*,*t*) = *K*(*s*,*t*) – Ks(*s*)Kt(*t*).

### 3.3. Transmission Dynamics Assessment

To evaluate local transmission potential beyond simple case counts, the intensity of spatiotemporal interaction (STK value) within 3 km and 6 days was analyzed. Based on the distribution of STK values across waves, epidemics were classified into “high interaction” and “low interaction” types. These contrasting transmission regimes are visualized using heatmaps, where higher interaction intensity is concentrated within short spatiotemporal windows during high‐interaction waves (Supporting Information 5: Figure S3). In contrast, low‐interaction waves show comparatively weaker and more diffuse interaction patterns, consistent with less cohesive short‐range spatiotemporal clustering.

Among the high‐interaction type, the 5th wave recorded an STK value of 7.30 × 10^11^, indicating the strongest short‐range spatiotemporal interaction under the present metric. Notably, the 11th wave involved only 32 cases, yet its STK value (4.67 × 10^11^) ranked second highest, suggesting that even relatively small outbreaks can exhibit highly concentrated transmission potential when cases occur in spatially dense settings.

Among the low‐interaction type, the 8th and 12th waves recorded low STK values. Particularly, the 12th wave maintained low interaction intensity despite its geographically confined diffusion pattern, implying that sporadic introductions and intermittent occurrences may have been more prominent than sustained local amplification within the affected region.

## 4. Discussion

This study suggests that the spatiotemporal clustering pattern of HPAI epidemics in South Korea shifted from a more clustered form to a more sporadic form around 2017. In post‐2017 epidemics, the frequency of strong STPSS clusters and high KDE maxima generally declined, and STK values were often lower than those observed during the major pre‐2017 epidemic waves. These pattern changes are temporally consistent with strengthened control measures after the 2016–2017 epidemic [28, 35], but they should be interpreted cautiously because this ecological analysis cannot separate policy effects from other time‐varying epidemiological and surveillance‐related factors.

This post‐2017 structural shift coincides with a multilayered reinforcement of Korea’s HPAI governance and field implementation capacity. First, the government reorganized the national “control tower” by establishing a dedicated Animal Health Policy Bureau in August 2017, separating disease control functions from livestock promotion and strengthening role clarity and accountability in the outbreak response. Second, implementation capacity was expanded through increased fiscal inputs, enabling targeted interventions such as the winter duck farming restriction program in high‐risk areas (introduced in November 2017 with dedicated compensation budgets) and mandatory CCTV installation for poultry farms (from May 2018 with governmental financial support). Third, operational surge capacity and perimeter control were reinforced through the expansion of joint disinfection teams and increased support for control posts and disinfection expenditures, facilitating faster containment and reduced between‐farm connectivity during epidemics [36].

An additional consideration is the expansion of active surveillance overtime in South Korea, particularly in duck‐related production systems and live bird market settings. National surveillance and diagnostic efforts have intensified substantially overtime, which may have increased surveillance sensitivity and case ascertainment across epidemic waves, especially for subclinical or mildly affected premises [37, 38]. Therefore, the observed post‐2017 shift may reflect not only changes in transmission conditions but also changes in detection intensity.

The strong spatiotemporal clusters observed before 2017, particularly during the 5th and 6th waves, suggest that once the virus was introduced into a specific region, it rapidly amplified and spread along epidemiological networks connecting farms, such as vehicles, personnel, and logistics [39]. In contrast, the significant reduction in clusters and the sharp decline in STK values in post‐2017 epidemics support the interpretation that a policy package designed to sever connectivity, including Korean Animal Health Integrated System (KAHIS)–based livestock vehicle movement control, designated disinfection facilities, and temporary standstill orders, altered transmission conditions in the field [40]. However, rather than asserting a linear causal relationship where policy directly leads to reduction, it is more appropriate to present this interpretation as the observed pattern changes that coincide temporally with policy reinforcement and align theoretically with the expected effects of network disruption [41].

This interpretation of cluster suppression through the severance of connectivity is consistent with overseas epidemic cases. For instance, studies of the 2016–2017 H5N8 epidemic in France found that spatial and administrative control measures influenced cluster formation in specific regions and that long‐distance movements within duck production systems potentially facilitated transmission. These findings suggest that in South Korea as well, movement and trading networks between farms, rather than simple geographic density alone, may contribute to the persistence or reconfiguration of outbreak patterns [42]. Therefore, the more sporadic pattern observed after 2017 may reflect a shift toward a phase in which primary introductions from wild birds to individual farms are relatively more prominent. However, it is more conservative to interpret this shift not as a complete interruption of subsequent secondary and tertiary spread but rather as a weakening of the length and cohesion of transmission chains. At the same time, this interpretation should be bound by the methodological scope of the present study. Because our analyses are based on distance‐defined spatial relationships, they are more informative for localized clustering and neighborhood‐scale interactions than for long‐distance transmission mediated by integrated production systems, transport routes, or trading networks. Therefore, the present findings should be interpreted as evidence of changes in the local and regional clustering structure, not as a full reconstruction of the complete transmission network.

The recurrent confirmation of the role of duck farms in the initial spread of epidemics is critical from a policy perspective [8]. Domestic risk prediction and case–control studies report that duck rearing density, proximity to live bird markets (LBMs), and farm scale and density significantly increase outbreak risk [43, 44]. From an industrial structure standpoint, factors such as long‐distance movement associated with vertical integration and the avoidance of reporting have been identified as accelerating horizontal transmission between farms [45]. This aligns with findings from the 2015 H5N2 epidemic in the United States, where anthropogenic and geographic factors, such as road density, were reported as key drivers of the spread [46]. Furthermore, phylogenetic and integrated analyses of domestic H5N8 and H5N6 viruses suggest maintenance within duck populations, interspecies transmission pathways, and increased local transmission potential in duck‐dense areas [47, 48]. This supports the view that ducks function not merely as a high‐risk species but as an amplifier in which industrial networks and biological characteristics interact. This interpretation is also supported by the STPSS results, where statistically significant clusters frequently involved duck premises in major epidemics. For example, multiple clusters in the 5th wave (H5N8) included meat and/or breeder ducks, and the primary cluster in the 6th wave (H5N6) was largely composed of meat ducks (Supporting Information 1: Table S1).

The potential of ducks as silent carriers has been consistently highlighted in experimental and review literature [49]. Pathogenicity studies using domestic isolates from 2020 to 2021 also reported that ducks can survive without clinical symptoms while shedding the virus for a certain period and transmitting it to cohabiting individuals [11]. Consequently, there is a possibility that ducks act as regional hubs when combined with duck farming environments during the early stages of an epidemic [50]. To address this risk of early amplification, the duck winter rest period policy was introduced in November 2017 as a targeted intervention to restrict duck farming in high‐risk areas during the winter season and provide compensation [14]. Furthermore, causal inference based on Bayesian structural time series (BSTS) analysis reported that weekly HPAI cases decreased by an average of 75% compared to counterfactual predictions after policy implementation, suggesting that the policy mechanism of reducing the initial amplification window is quantitatively supported [14]. In addition to prior evidence of reduced incidence, this study confirms spatial and structural changes, namely, a reduction in clusters and a decline in STK‐based spatiotemporal interaction intensity, after 2017. This provides further consistent evidence that strengthened control strategies may have weakened the cohesion of interfarm transmission chains.

However, despite this structural transition toward more sporadic patterns, the detection of intermittent high‐risk signals after 2017 suggests a residual risk in the blind spots of disease control. A prime example is the 11th wave; although the total number of cases was relatively low (*N* = 32), the STK value was recorded at 4.67 × 10^11^, the second‐highest value recorded. This paradoxical finding implies that while strengthened policies may have substantially reduced long‐distance spread and large‐scale cluster formation, the “local transmission potential” within high‐density farming areas remains explosive once the virus is introduced. This indicates that a low number of cases does not necessarily imply a low transmission risk. Therefore, current policies need to expand beyond regulating large‐scale commercial networks to include targeted management of these high‐interaction‐risk zones.

This residual risk is linked to existing discussions that biosecurity and vehicle control may be relatively vulnerable in small‐scale farms (backyards), free‐range farming systems, and mixed farming systems [51]. This concern is consistent with our STPSS cluster composition results, which included premises categorized as “mixed rearing” within statistically significant clusters (e.g., the primary cluster in the 3rd wave; Supporting Information 1: Table S1). Future policies should extend beyond regulating large‐scale farms to include fact‐finding surveys of blind spots and customized management measures, such as education, infrastructure improvement, movement management, and reporting incentives.

This study has some limitations. First, as a wave‐level ecological analysis based on outbreak locations, this study cannot directly establish the causality between observed pattern changes and policy reinforcement. In addition, the analysis did not formally adjust for major time‐varying confounders across the study period, including viral subtype, wild‐bird‐related introduction pressure, surveillance intensity, and changes in farm density or production structure. Second, due to the constraints of the cylindrical scan window, irregular diffusion patterns may not have been fully captured [52]. Incorporating a spatiotemporal adaptive kernel approach that reflects dynamic background populations in future studies could improve precision [53]. Nevertheless, the ability of STPSS to identify interaction patterns even under the spatiotemporal uncertainty of data supports the interpretability and robustness of this study’s findings [21].

Overall, our 20‐year analysis indicates a clear shift before and after 2017 from clustered epidemics to more sporadic patterns, accompanied by reduced cluster frequency and lower STK–based spatiotemporal interaction intensity. These findings are consistent with the possibility that strengthened control policies contributed to weakening the cohesion of interfarm transmission chains while highlighting the need for risk‐based surveillance targeting residual high‐risk clusters and blind‐spot farms [54, 55]. Furthermore, optimization strategies that account for spatial heterogeneity, such as risk‐based adjustment of culling radii, could simultaneously improve cost effectiveness and social acceptance [56].

## 5. Conclusion

This study provides quantitative evidence that the spatiotemporal pattern of HPAI in South Korea underwent a significant structural shift around 2017, coinciding with the reinforcement of control policies. The transition from large‐scale clustered epidemics to more sporadic occurrences, coupled with an overall decline in spatiotemporal interaction intensity, was temporally consistent with reinforced control policies after 2017 and may also reflect concurrent changes in surveillance intensity and other epidemiological conditions. Therefore, these findings should be interpreted as evidence of changing outbreak structure rather than as direct proof of the policy effect alone.

Nevertheless, the persistence of high interaction values during minor epidemics (e.g., the 11th wave) and the localized circular spread observed in the recent 12th wave indicate that the risk has not been eliminated but rather contained. The virus still retains the capacity for rapid local amplification in vulnerable areas. Therefore, future control strategies should evolve from uniform regulation toward precision management. We recommend implementing risk‐based surveillance that specifically targets residual high‐risk clusters and blind spots identified in this study, along with dynamic adjustment of control measures based on real‐time spatiotemporal risk assessment.

## Author Contributions

Sung Dae Park conceived the study, analyzed the data, and drafted the manuscript. Ji Hyun Son participated in data curation and interpretation of the results. Dae Sung Yoo supervised the study and revised the manuscript.

## Funding

This work was supported by Korea Institute of Planning and Evaluation for Technology in Food, Agriculture and Forestry (IPET) through the High‐Risk Animal Infectious Disease Control Technology Development Project, funded by the Ministry of Agriculture, Food and Rural Affairs (MAFRA) (Grant RS‐2024‐00399814).

## Disclosure

All authors have read and approved the final manuscript.

## Ethics Statement

This study involved a retrospective analysis of secondary data on animal disease outbreaks and did not include experimental research involving humans or animals.

## Consent

The authors have nothing to report.

## Conflicts of Interest

The authors declare no conflicts of interest.

## Supporting Information

Additional supporting information can be found online in the Supporting Information section.

## Supporting information


**Supporting Information 1** Table S1. Characteristics of statistically significant spatiotemporal clusters of HPAI detected by STPSS (2003–2025). This table provides detailed epidemiological data for 43 significant clusters, including center coordinates, radius, observed/expected case ratios, and affected poultry species.

**Supporting Information 2** Table S2. Sensitivity analysis of KDE maxima across epidemic waves using Gaussian kernel bandwidths of 5, 10, and 15 km.

**Supporting Information 3** Figure S1. Spatiotemporal distribution of statistically significant HPAI clusters in South Korea (2003–2025). This figure visualizes the spatial extent and relative timing of clusters for each epidemic wave as detected by the space‐time permutation scan statistic (STPSS).

**Supporting Information 4** Figure S2. Macrospatial patterns of HPAI epidemic waves: kernel density estimation (KDE) and standard deviational ellipses (SDE). This includes density maps highlighting hotspots (KDE) and ellipses representing the directional distribution and centrality (SDE) of outbreaks.

**Supporting Information 5** Figure S3. Heatmaps of spatiotemporal interaction intensity, *D*(*s*,*t*), derived from the space–time *K*‐function for major HPAI epidemic waves. These heatmaps illustrate the strength of local farm‐to‐farm transmission interaction within defined distance and time intervals.

## Data Availability

The case data analyzed in this study were obtained from the KAHIS and internal records of the MAFRA. Publicly accessible components can be retrieved via KAHIS, while access to nonpublic case‐level data may be subject to institutional restrictions. Administrative boundary data are available from the Statistical Geographic Information Service (SGIS). Aggregated data supporting the findings and the analysis code are available from the corresponding author upon reasonable request.
