# Supplementary material for: From Clustered to Sporadic: Structural Shifts in the Spatiotemporal Dynamics of HPAI Following the 2017 Policy Reinforcement in South Korea (2003–2025)
Source: Transbound Emerg Dis. 2026 Jul 7;2026:5747471. doi: 10.1155/tbed/5747471 (PMC13340132; doi:10.1155/tbed/5747471)
Supplement: Supplementary file 1 — Supporting Information 1 Table S1. Characteristics of statistically significant spatiotemporal clusters of HPAI detected by STPSS (2003–2025). This table provides detailed epidemiological data for 43 significant clusters, including center coordinates, radius, observed/expected case ratios, and affected poultry species. [file TBED-2026-5747471-s002.docx]

**Appendix Figure 2.** Macro-spatial patterns of HPAI epidemic waves: kernel density estimation (KDE) and standard deviational ellipses (SDE).

| **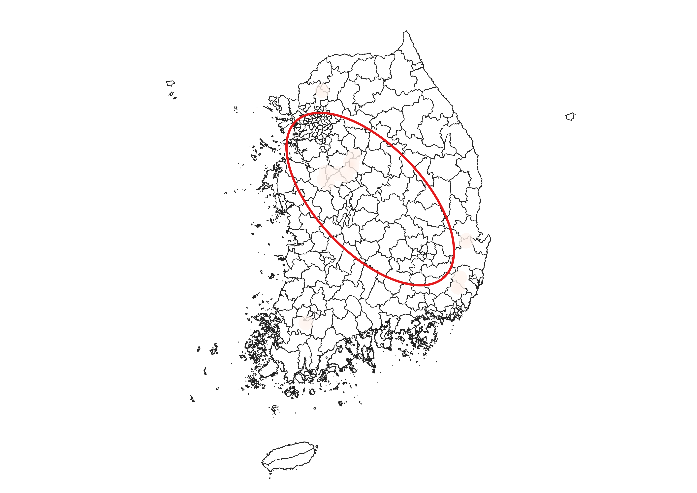** | 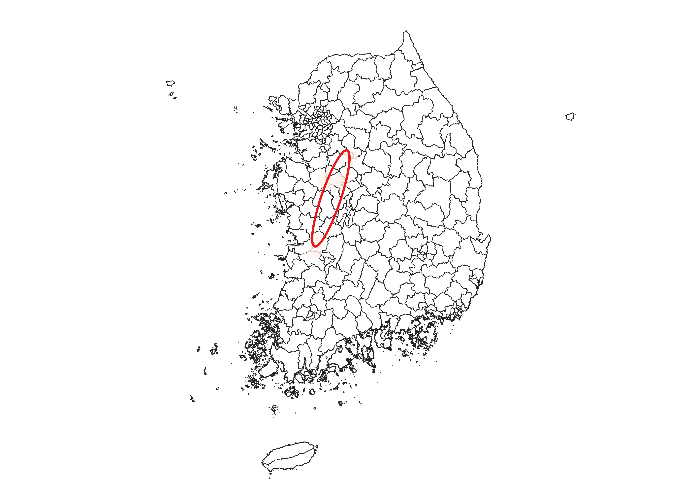 |
| --- | --- |
| **<1st Wave: 2003.12.10 –2004.3.20>**  **19 cases outbreak, 1 cluster formed** | **<2nd Wave: 2006.11.22 –2007.3.20>**  **13 cases outbreak, no cluster formed** |
| **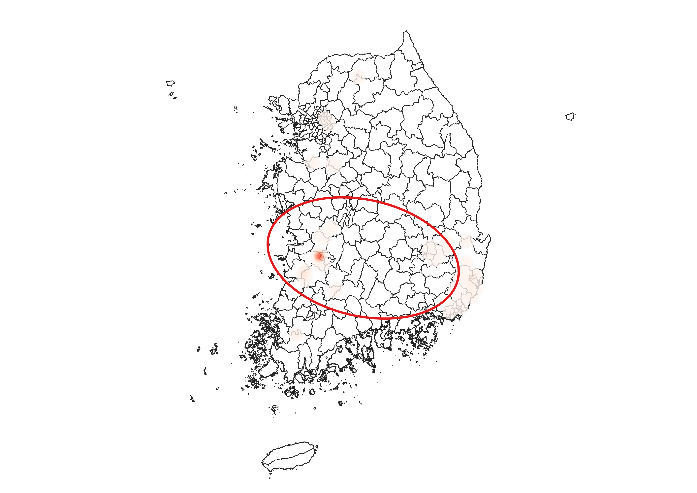** | **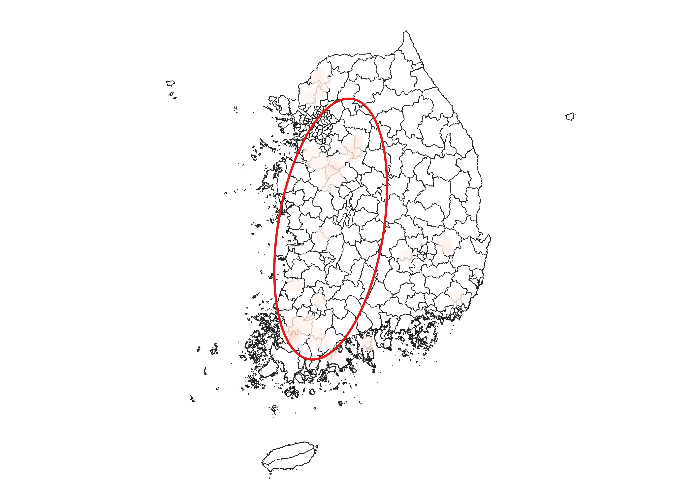** |
| **<3rd Wave: 2008.4.1–2008.5.24>**  **98 cases outbreak, 4 clusters formed** | **<4th Wave: 2010.12.29–2011.5.21>**  **91 cases outbreak, 5 clusters formed** |
| **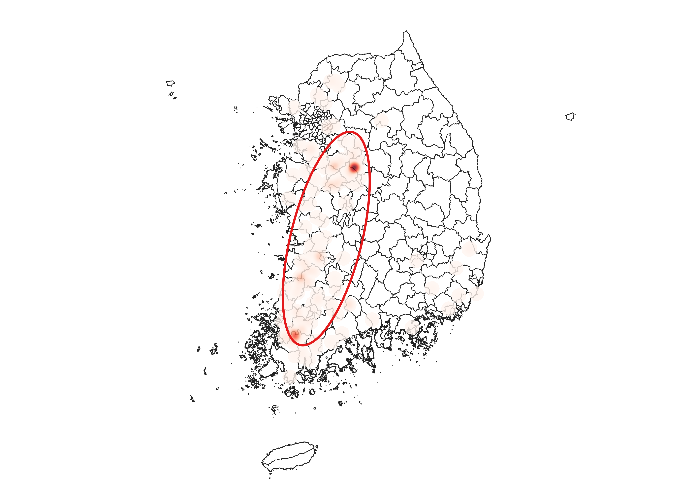** | **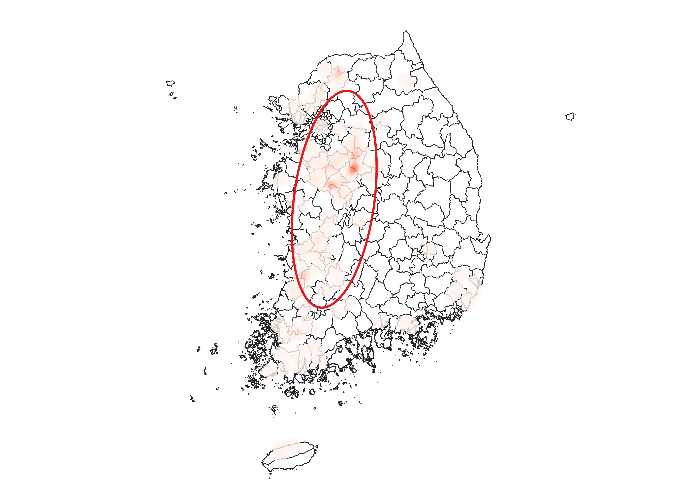** |
| **<5th Wave: 2014.01.16–2016.04.05>**  **393 cases outbreak, 17 clusters formed** | **<6th Wave: 2016.11.16–2017.06.19>**  **419 cases outbreak, 13 clusters formed** |

| **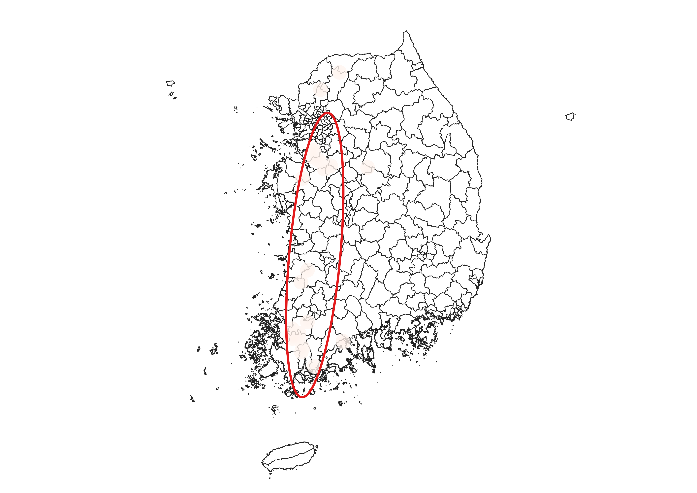** | 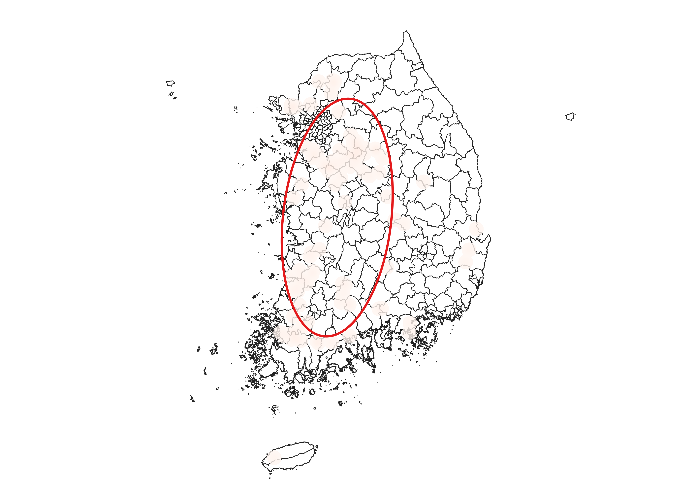 |
| --- | --- |
| **<7th Wave: 2017.11.17–2018.03.17>**  **22 cases outbreak, no cluster formed** | **<8th Wave: 2020.11.26–2021.04.06>**  **109 cases outbreak, no cluster formed** |
| **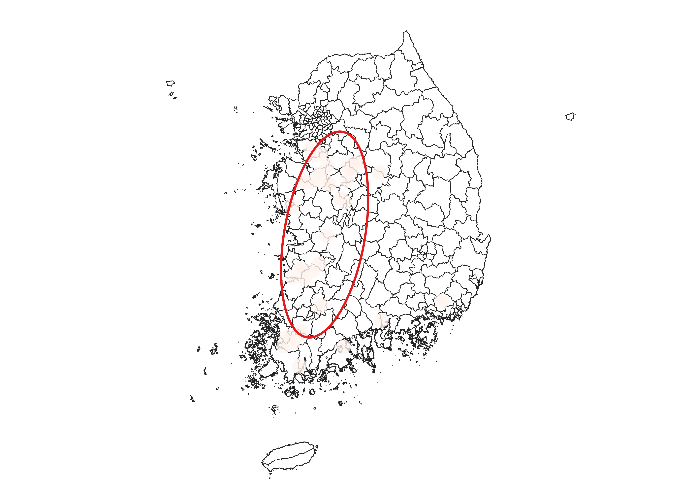** | **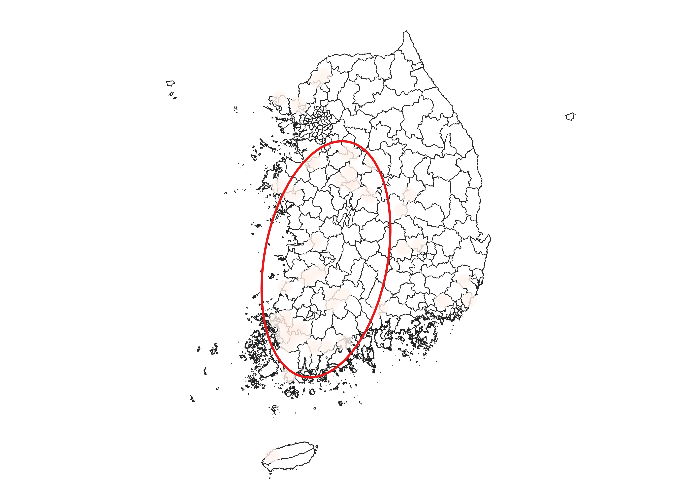** |
| **<9th Wave: 2021.11.08–2022.04.07>**  **47 cases outbreak, no cluster formed** | **<10th Wave: 2022.10.17–2023.04.14>**  **75 cases outbreak, 2 clusters formed** |
| **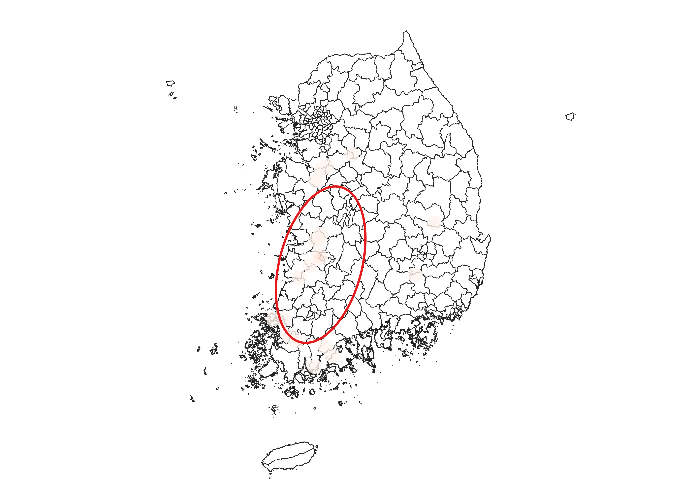** | **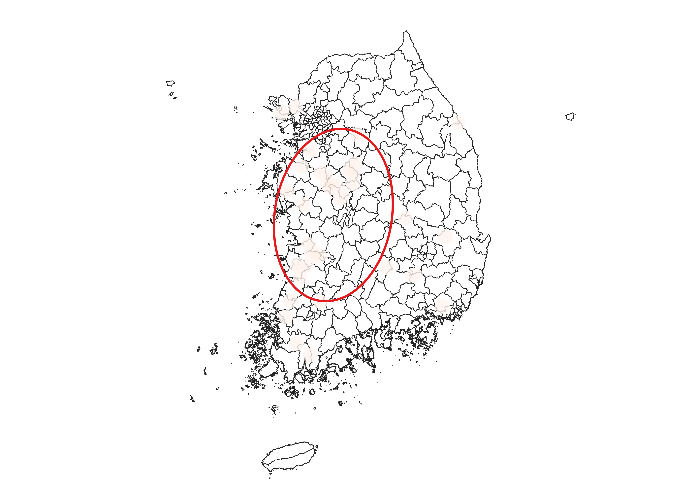** |
| **<11th Wave: 2023.12.03–2024.05.22>**  **32 cases outbreak, no cluster formed** | **<12th Wave: 2024.10.29–2025.06.27>**  **49 cases outbreak, 1 cluster formed** |

**Note:** Red areas represent high-density hotspots based on KDE. Ellipses indicate the directional distribution of outbreaks (1 standard deviation). Waves 1–11 generally show predominantly linear spread (high eccentricity) along the north–south axis. Wave 12 shows a more circular pattern (lower eccentricity), suggesting that spread was relatively more geographically confined to a specific region. This pattern is temporally consistent with strengthened movement controls but should not be interpreted as direct evidence of policy effect alone. **Base map source:** Statistical Geographic Information Service (SGIS)
