# Supplementary material for: From Clustered to Sporadic: Structural Shifts in the Spatiotemporal Dynamics of HPAI Following the 2017 Policy Reinforcement in South Korea (2003–2025)
Source: Transbound Emerg Dis. 2026 Jul 7;2026:5747471. doi: 10.1155/tbed/5747471 (PMC13340132; doi:10.1155/tbed/5747471)
Supplement: Supplementary file 2 — Supporting Information 2 Table S2. Sensitivity analysis of KDE maxima across epidemic waves using Gaussian kernel bandwidths of 5, 10, and 15 km. [file TBED-2026-5747471-s003.docx]

**Appendix Figure 1.** Spatiotemporal distribution of statistically significant HPAI clusters in South Korea (2003–2025)

| 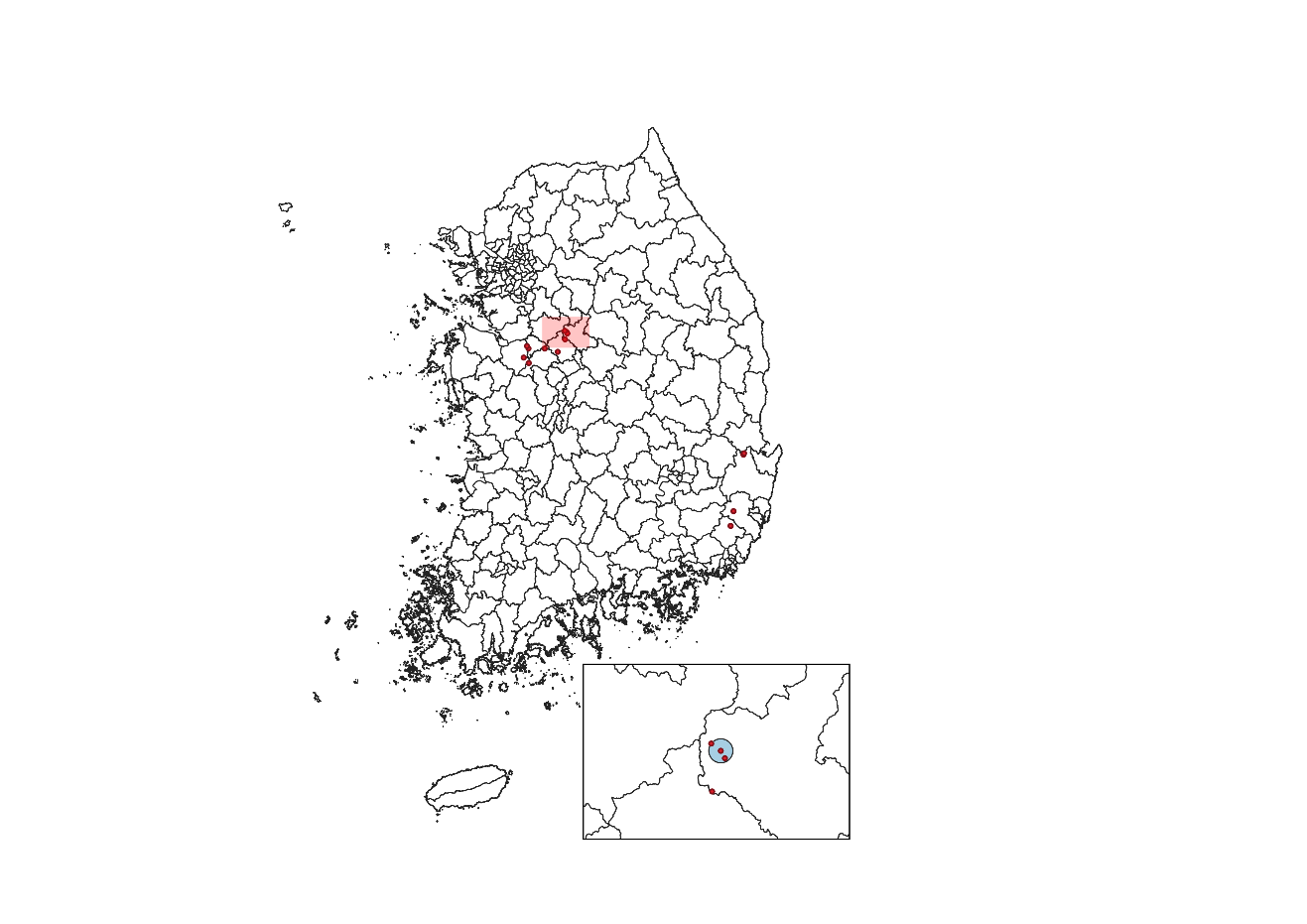 | 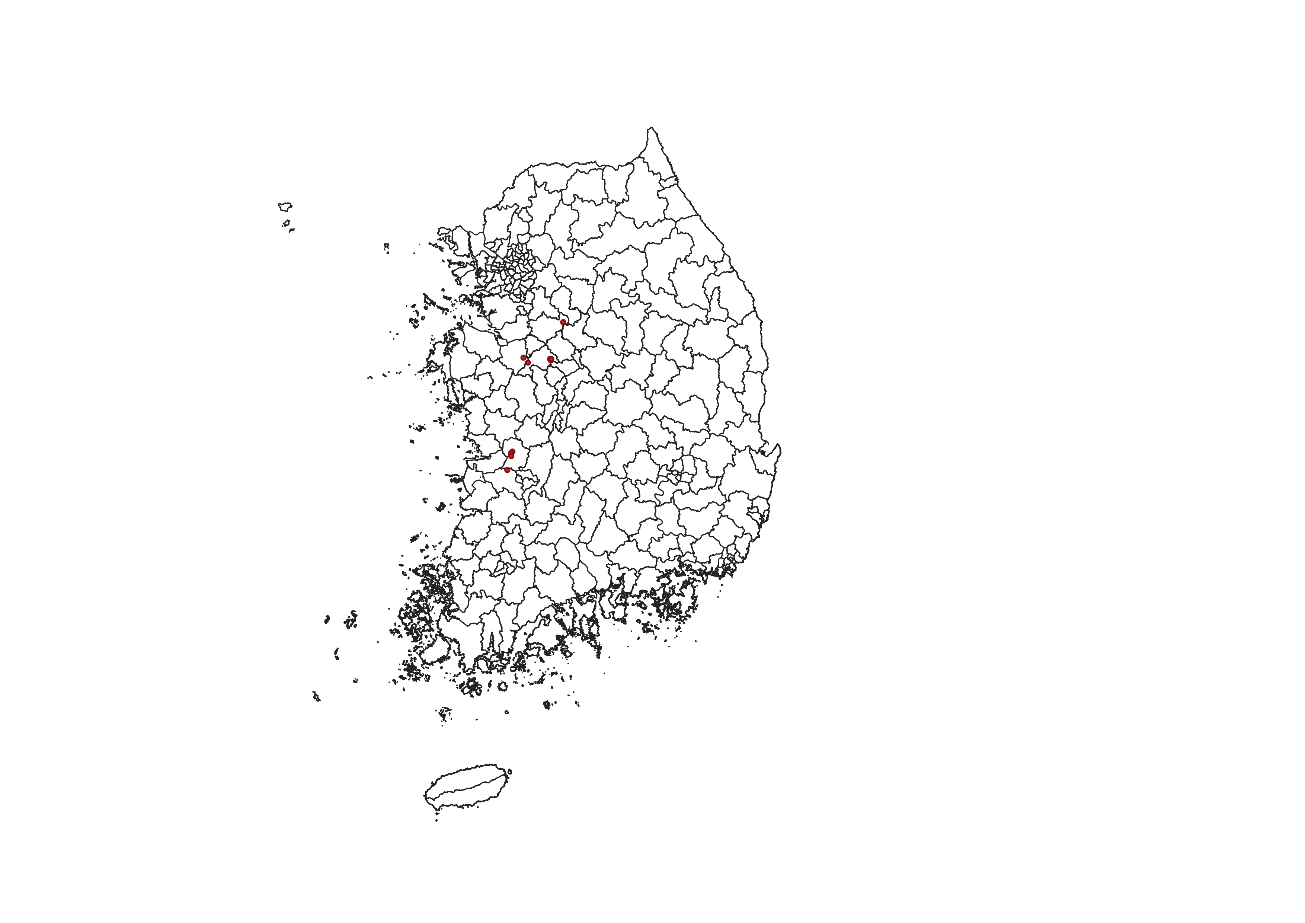 |
| --- | --- |
| **<1st Wave: 2003.12.10–2004.3.20>**  **19 cases outbreak, 1 cluster formed** | **<2nd Wave: 2006.11.22 –2007.3.20>**  **13 cases outbreak, no cluster formed** |
| 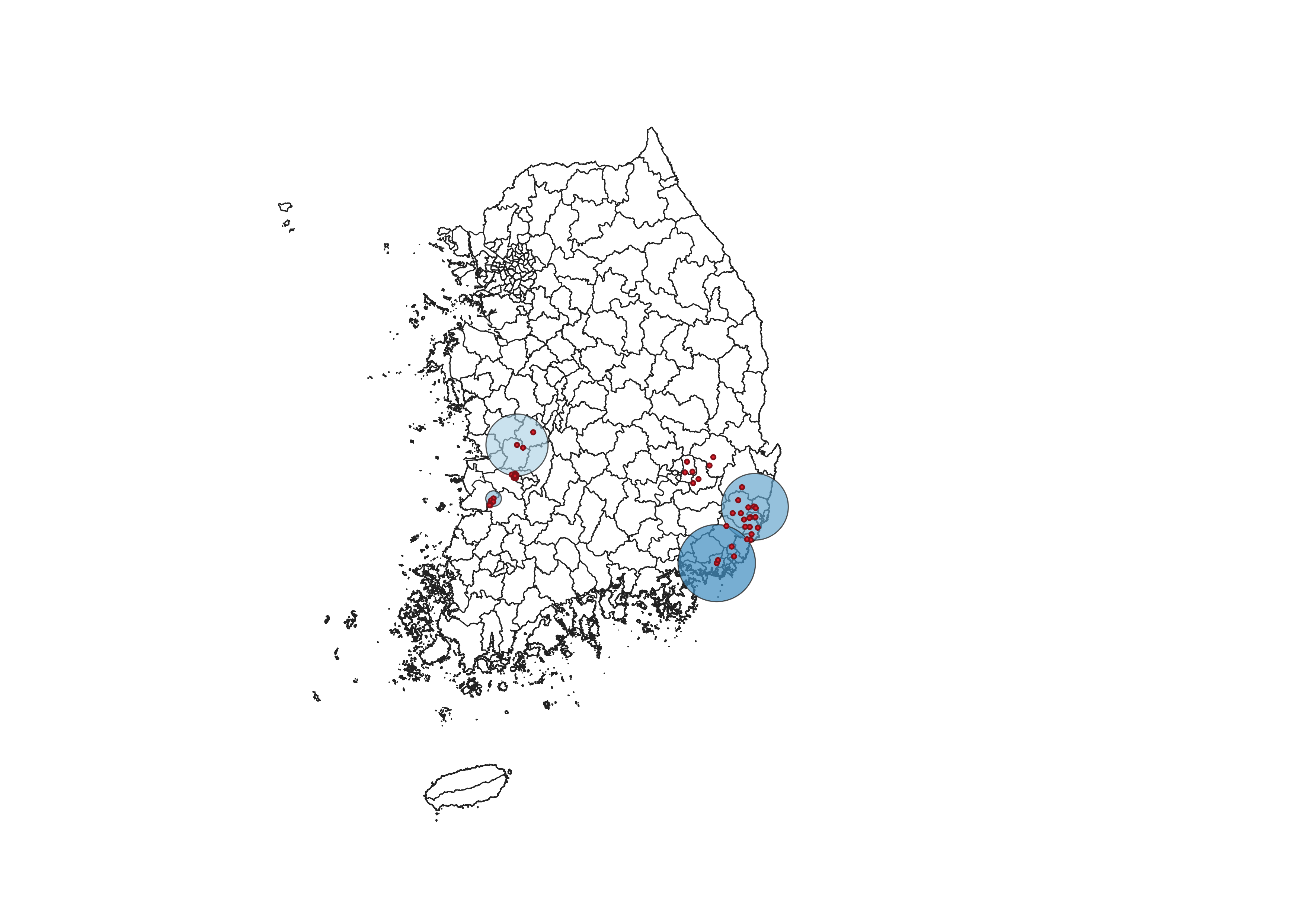 | 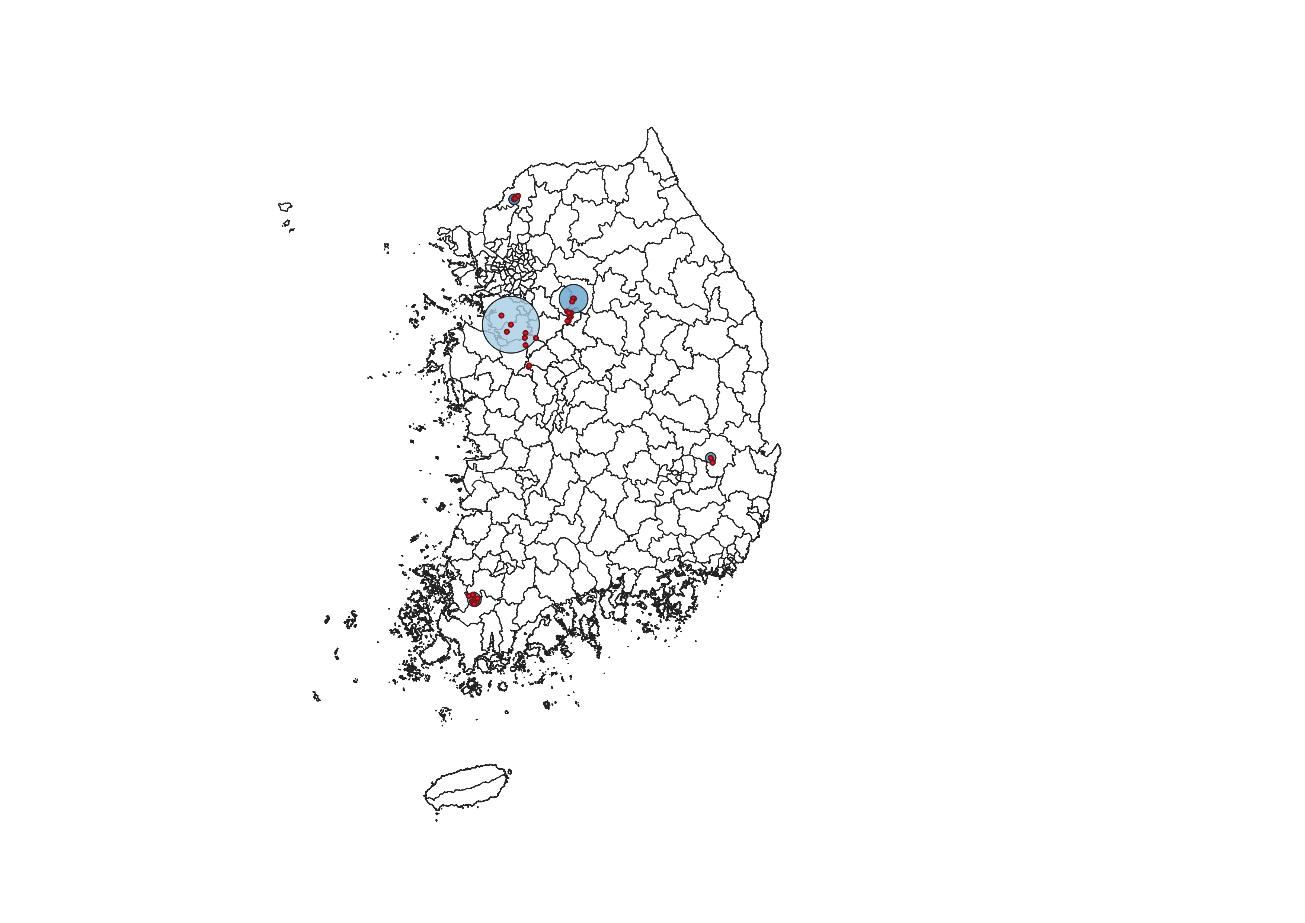 |
| **<3rd Wave: 2008.4.1–2008.5.24>**  **98 cases outbreak, 4 clusters formed** | **<4th Wave: 2010.12.29–2011.5.21>**  **91 cases outbreak, 5 clusters formed** |

| 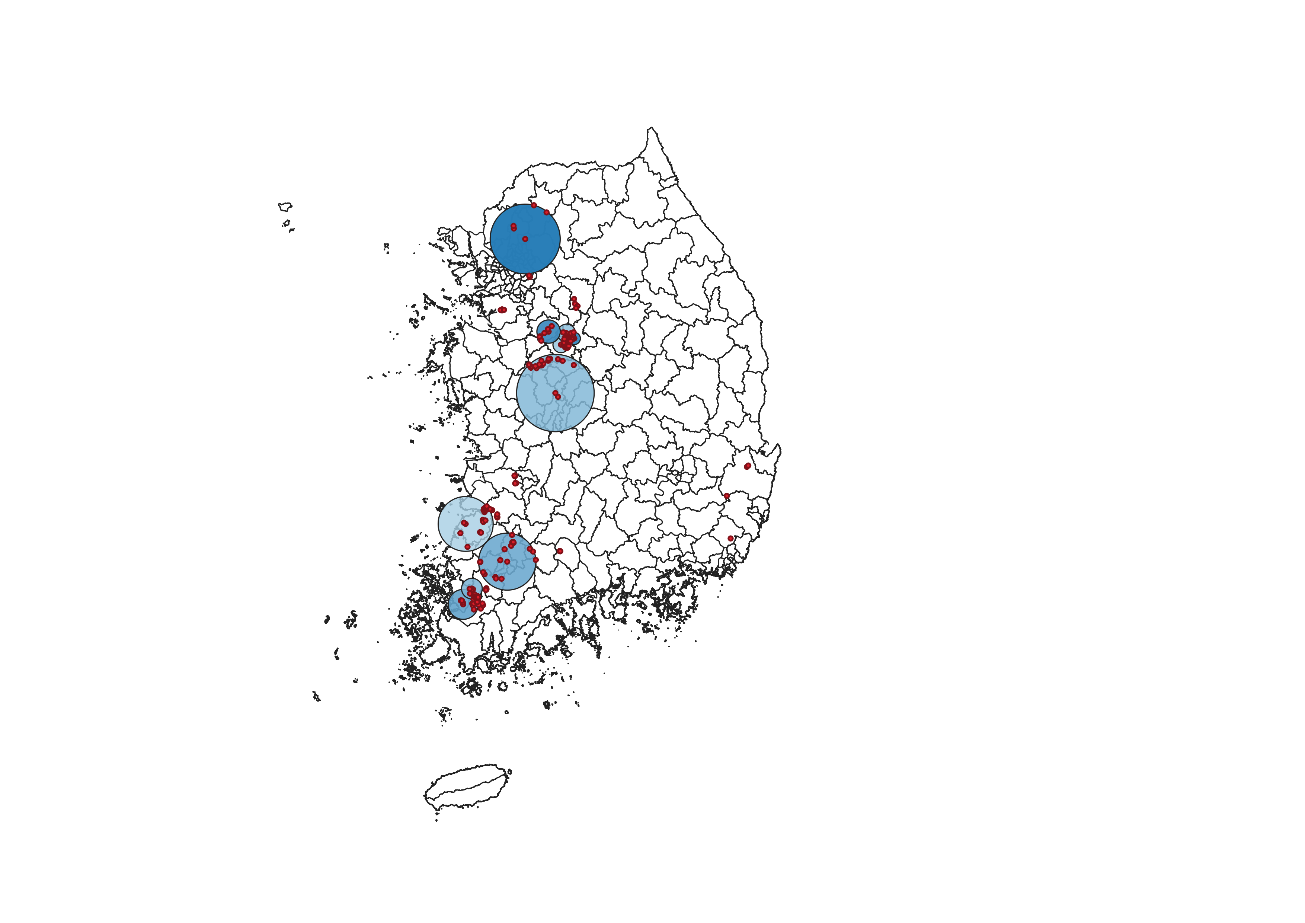 | 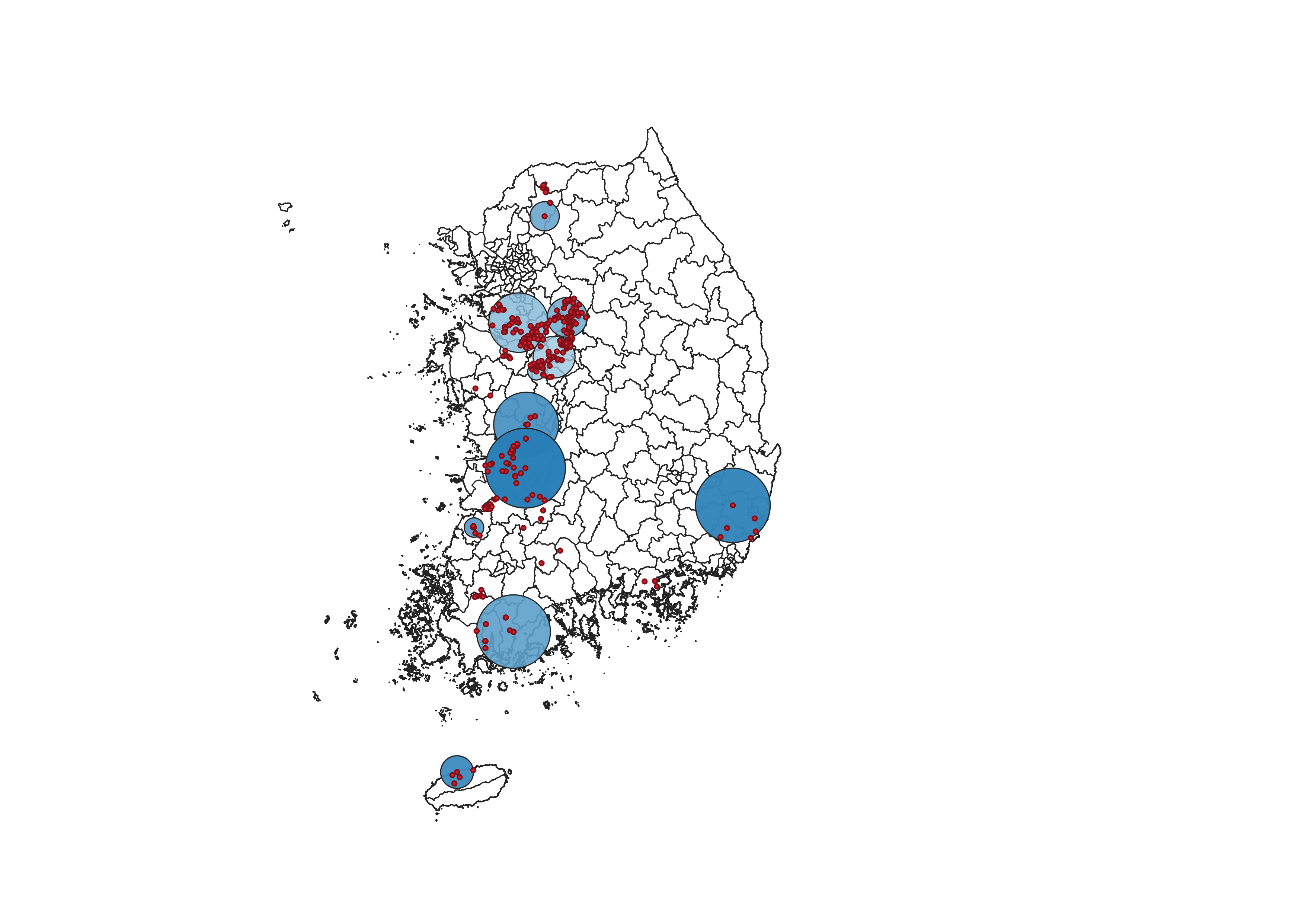 |
| --- | --- |
| **<5th Wave: 2014.01.16–2016.04.05>**  **393 cases outbreak, 17 clusters formed** | **<6th Wave: 2016.11.16–2017.06.19>**  **419 cases outbreak, 13 clusters formed** |
| 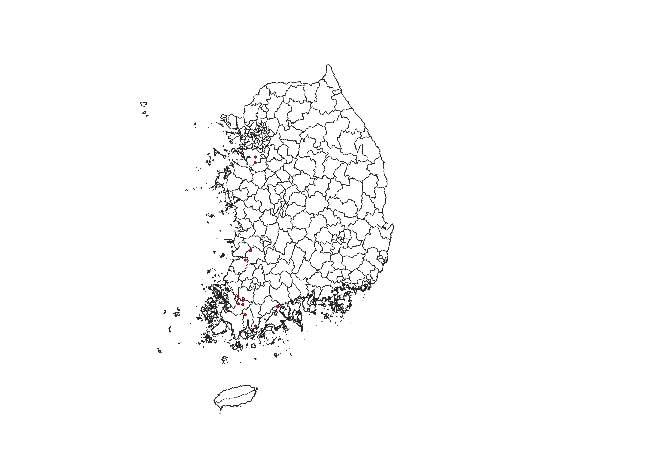 | 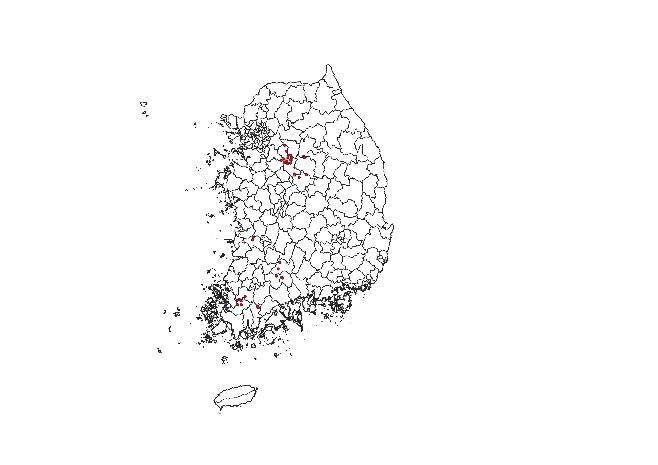 |
| **<7th Wave: 2017.11.17–2018.03.17>**  **22 cases outbreak, no cluster formed** | **<8th Wave: 2020.11.26–2021.04.06>**  **109 cases outbreak, no cluster formed** |

| 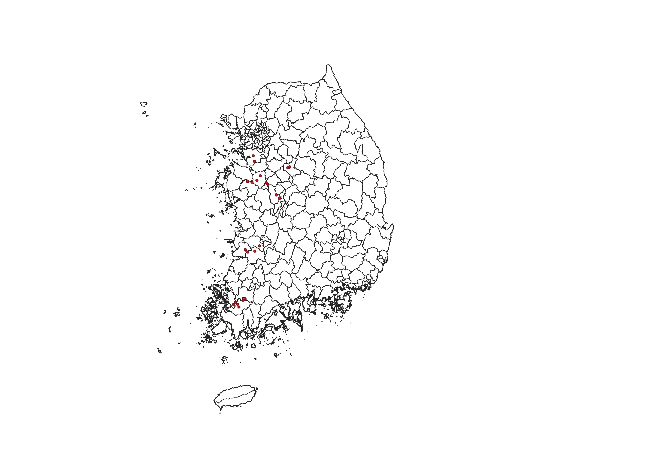 | 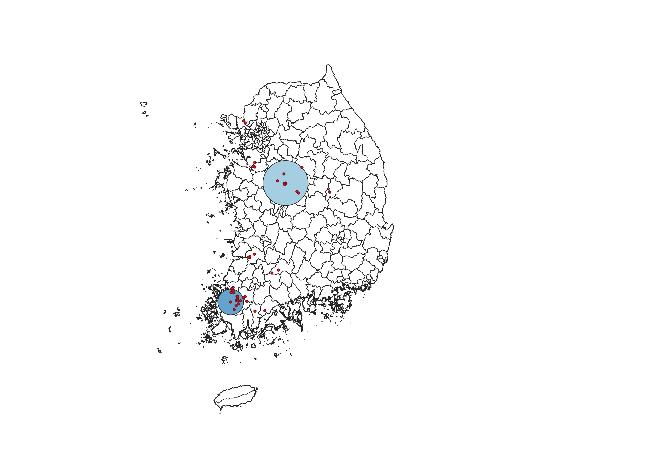 |
| --- | --- |
| **<9th Wave: 2021.11.08–2022.04.07>**  **47 cases outbreak, no cluster formed** | **<10th Wave: 2022.10.17–2023.04.14>**  **75 cases outbreak, 2 clusters formed** |
| 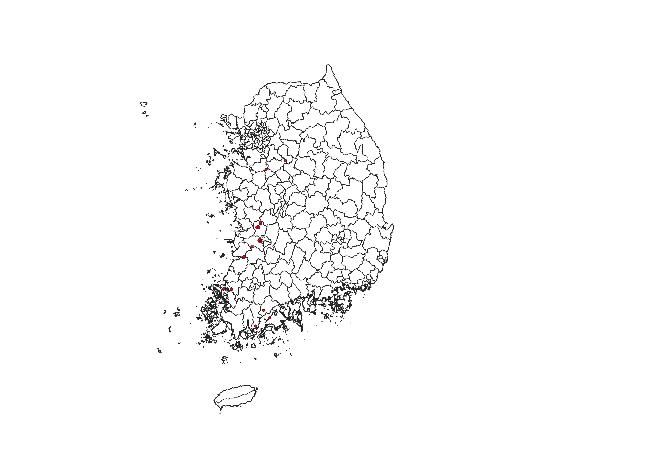 | 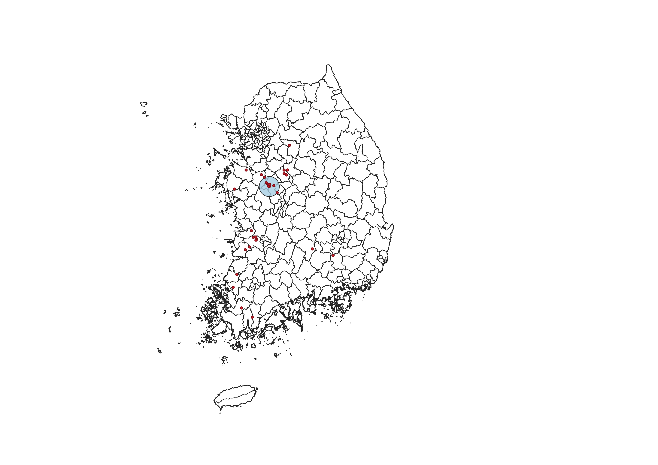 |
| **<11th Wave: 2023.12.03–2024.05.22>**  **32 cases outbreak, no cluster formed** | **<12th Wave: 2024.10.29–2025.06.27>**  **49 cases outbreak, 1 cluster formed** |

**Note:** Circles indicate the spatial extent of statistically significant clusters detected using the space–time permutation scan statistic (p < 0.05). Circle shading represents the relative timing of cluster occurrence within each wave (lighter = earlier, darker = later). No significant clusters were detected in Waves 2, 7, 8, 9, and 11, consistent with less sustained wave-level clustering and relatively more dispersed occurrence patterns. **Base map source:** Statistical Geographic Information Service (SGIS).
