# Supplementary material for: From Clustered to Sporadic: Structural Shifts in the Spatiotemporal Dynamics of HPAI Following the 2017 Policy Reinforcement in South Korea (2003–2025)
Source: Transbound Emerg Dis. 2026 Jul 7;2026:5747471. doi: 10.1155/tbed/5747471 (PMC13340132; doi:10.1155/tbed/5747471)
Supplement: Supplementary file 3 — Supporting Information 3 Figure S1. Spatiotemporal distribution of statistically significant HPAI clusters in South Korea (2003–2025). This figure visualizes the spatial extent and relative timing of clusters for each epidemic wave as detected by the space‐time permutation scan statistic (STPSS). [file TBED-2026-5747471-s004.docx]

**Appendix Figure 3.** Heatmaps of spatiotemporal interaction intensity, D(s,t), derived from the space–time K-function for major HPAI epidemic waves.

| 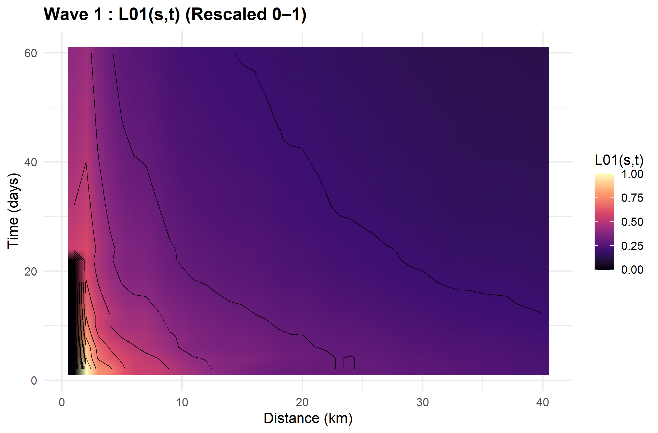 | 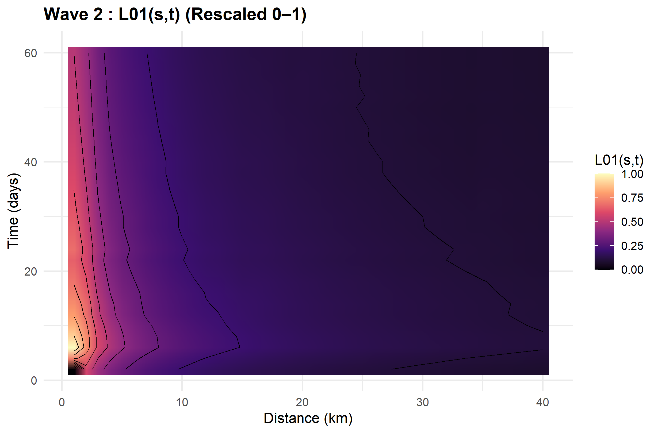 |
| --- | --- |
| 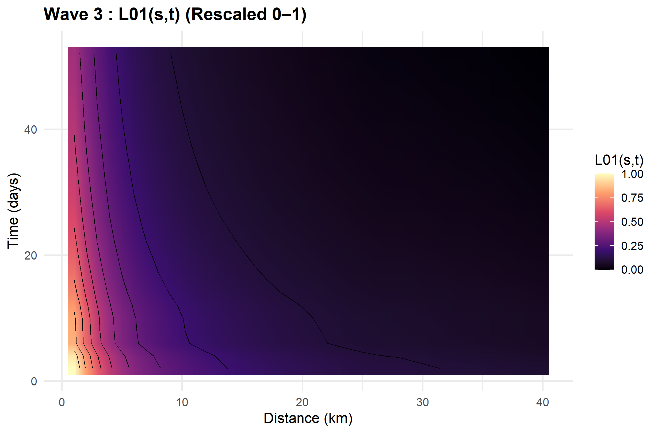 | 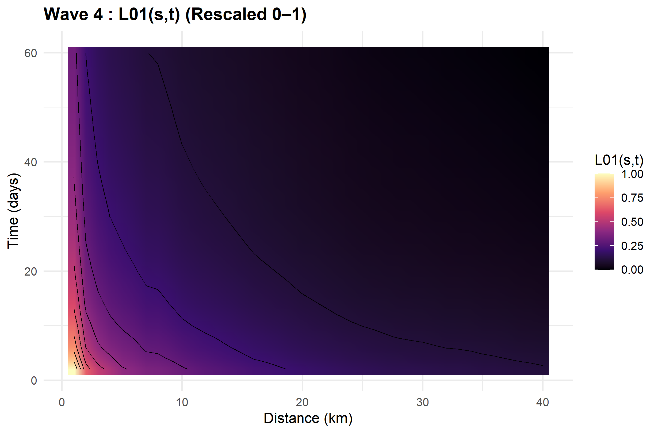 |
| 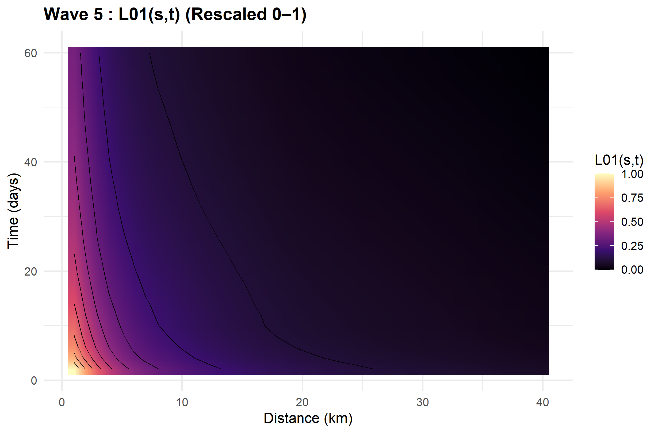 | 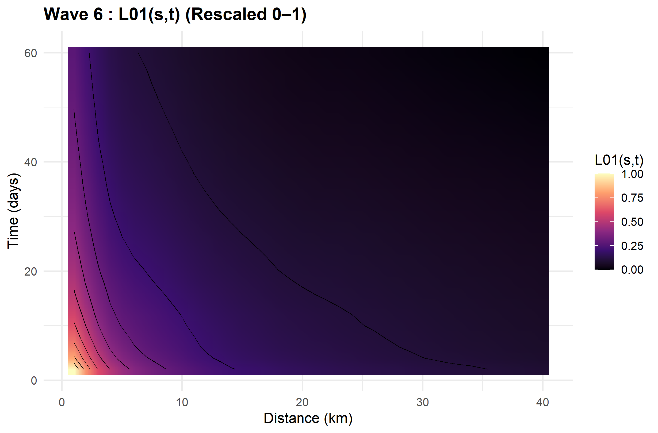 |
| 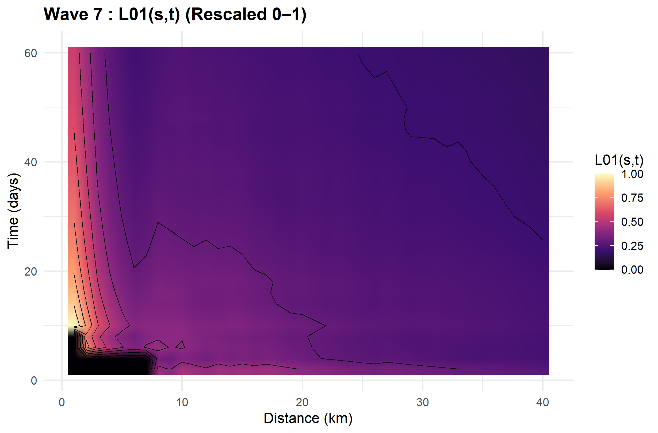 | 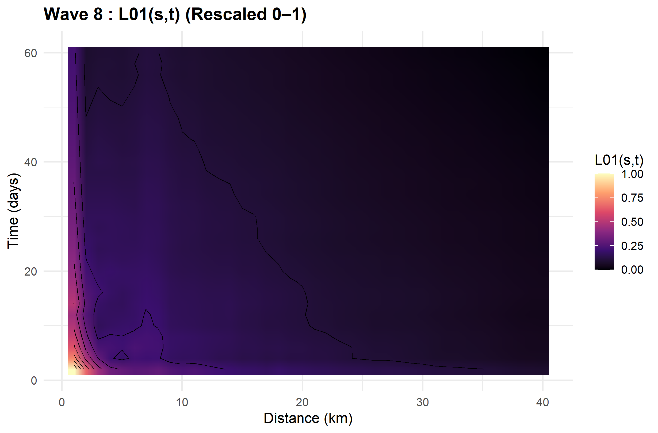 |
| 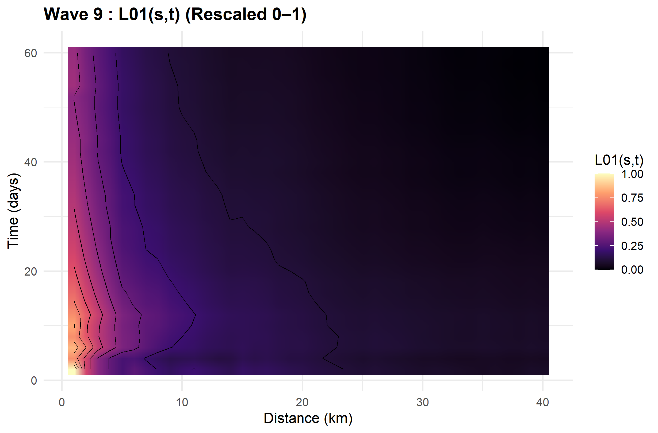 | 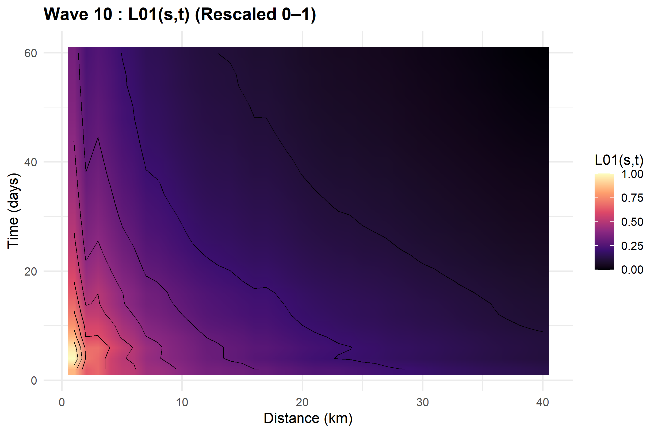 |
| 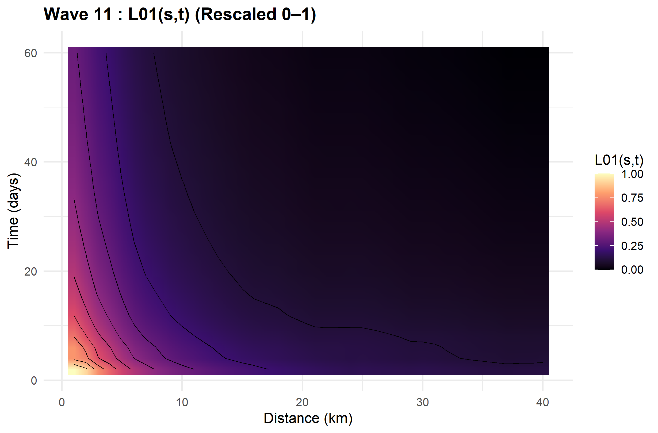 | 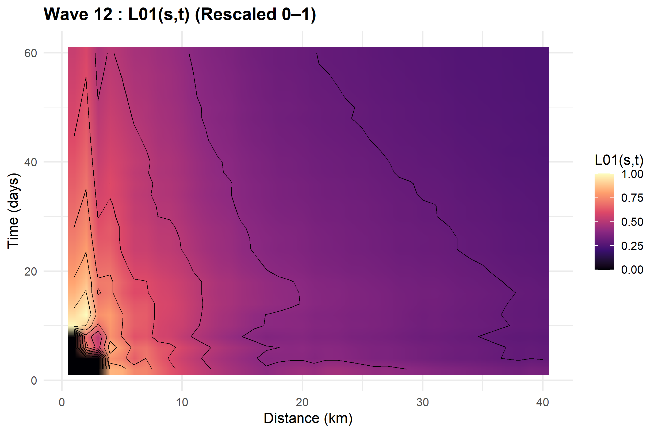 |

**Note:** The x-axis represents distance (km) and the y-axis represents time interval (days). Warmer colors indicate stronger spatiotemporal clustering (higher D[s,t]). For example, Wave 5 shows strong interaction (D[s,t] > 10^11^), whereas Wave 8 shows minimal interaction (D[s,t] < 10^10^), reflecting marked differences in short-range spatiotemporal interaction intensity rather than direct evidence of transmission sequence.
