# Supplementary material for: From Clustered to Sporadic: Structural Shifts in the Spatiotemporal Dynamics of HPAI Following the 2017 Policy Reinforcement in South Korea (2003–2025)
Source: Transbound Emerg Dis. 2026 Jul 7;2026:5747471. doi: 10.1155/tbed/5747471 (PMC13340132; doi:10.1155/tbed/5747471)
Supplement: Supplementary file 4 — Supporting Information 4 Figure S2. Macrospatial patterns of HPAI epidemic waves: kernel density estimation (KDE) and standard deviational ellipses (SDE). This includes density maps highlighting hotspots (KDE) and ellipses representing the directional distribution and centrality (SDE) of outbreaks. [file TBED-2026-5747471-s005.docx]

**Appendix Table 1.** Characteristics of statistically significant spatiotemporal clusters of HPAI detected by STPSS (2003–2025)

| Wave | Cluster  rank | Period | Center  coordinates | Radius  (km) | Observed  cases | Expected  cases | O/E  ratio | P-value | Subtype | Species |
| --- | --- | --- | --- | --- | --- | --- | --- | --- | --- | --- |
| 1st | 1 | 2003/12/10–12/16 | 36.99N, 127.48E | 1.87 | 3 | 0.47 | 6.33 | 0.0305 | H5N1 | Broiler breeder (1), breeder duck (1), layer (1) |
| 3rd | 1 | 2008/5/1–5/8 | 35.59N, 129.32E | 29.33 | 16 | 4.51 | 3.55 | 0.00017 | H5N1 | Korean native chicken (11), mixed rearing (5) |
|  | 2 | 2008/4/16–4/22 | 36.09N, 127.00E | 27.23 | 17 | 5.62 | 3.02 | 0.0012 | H5N1 | Layer (13), breeding duck (1), Broiler breeder (2),  Korean native chicken (1) |
|  | 3 | 2008/5/9–5/15 | 35.15N, 128.94E | 33.93 | 5 | 0.43 | 11.67 | 0.0024 | H5N1 | Korean native chicken (3), meat duck (1), layer (1) |
|  | 4 | 2008/4/3–4/10 | 35.67N, 126.78E | 6.85 | 8 | 1.71 | 4.67 | 0.0302 | H5N1 | Meat duck (7), Korean native chicken (1) |
| 4th | 1 | 2011/3/22–4/16 | 35.98N, 128.90E | 4.57 | 5 | 0.27 | 18.20 | 0.00015 | H5N1 | Layer (5) |
|  | 2 | 2011/5/15–5/21 | 38.04N, 126.96E | 4.65 | 5 | 0.27 | 18.20 | 0.00015 | H5N1 | Layer (3), broiler (2) |
|  | 3 | 2011/1/3–1/9 | 34.86N, 126.60E | 5.93 | 14 | 3.46 | 4.04 | 0.00022 | H5N1 | Meat duck (11), breeder duck (3) |
|  | 4 | 2011/2/17–2/27 | 37.25N, 127.56E | 12.56 | 7 | 0.77 | 9.10 | 0.00034 | H5N1 | Layer (3), meat duck (1), breeder duck (1),  Broiler breeder (1), quail (1) |
|  | 5 | 2011/1/25–2/10 | 37.05N, 126.94E | 24.97 | 6 | 0.62 | 9.75 | 0.0010 | H5N1 | Korean native chicken (2), meat duck (1), breeder duck (1),  Broiler breeder (1), pheasant (1) |
| 5th | 1 | 2014/1/16–1/24 | 35.46N, 126.51E | 24.12 | 22 | 1.65 | 13.34 | <0.0001 | H5N8 | Meat duck (19), breeder duck (3) |
|  | 2 | 2015/3/26–4/12 | 35.85N, 126.99E | 0.49 | 11 | 0.34 | 32.75 | <0.0001 | H5N8 | Layer (11) |
|  | 3 | 2014/2/1–2/7 | 36.89N, 127.43E | 7.03 | 15 | 0.99 | 15.08 | <0.0001 | H5N8 | Meat duck (13), breeder duck (2) |
|  | 4 | 2015/2/22–3/13 | 36.94N, 127.56E | 5.39 | 32 | 6.89 | 4.64 | <0.0001 | H5N8 | Meat duck (28), breeder duck (2), broiler breeder (2) |
|  | 5 | 2014/2/17–2/24 | 36.98N, 127.49E | 7.93 | 34 | 8.08 | 4.21 | <0.0001 | H5N8 | Meat duck (32), breeder duck (2) |
|  | 6 | 2014/9/24–10/12 | 34.83N, 126.49E | 13.18 | 16 | 1.66 | 9.66 | <0.0001 | H5N8 | Meat duck (12), Korean native chicken (3), breeder duck (1) |
|  | 7 | 2015/10/18–11/16 | 34.87N, 126.63E | 2.35 | 10 | 0.66 | 15.12 | <0.0001 | H5N8 | Meat duck (10) |
|  | 8 | 2014/3/10–4/6 | 34.95N, 126.58E | 9.08 | 15 | 1.93 | 7.77 | <0.0001 | H5N8 | Breeder duck (7), meat duck (7), layer (1) |
|  | 9 | 2014/12/20–12/26 | 37.43N, 127.12E | 1.30 | 5 | 0.06 | 78.60 | <0.0001 | H5N8 | Korean native chicken (5) |
|  | 10 | 2014/5/16–11/7 | 35.17N, 126.92E | 25.10 | 16 | 2.52 | 6.36 | <0.0001 | H5N8 | Meat duck (10), Korean native chicken (3), broiler (1)  Goose (1), environmental sample (1) |
|  | 11 | 2015/4/16–5/21 | 37.73N, 127.08E | 30.69 | 6 | 0.15 | 39.30 | <0.0001 | H5N8 | Korean native chicken (3), meat duck (2), broiler (1) |
|  | 12 | 2015/1/24–1/30 | 34.96N, 126.72E | 1.53 | 7 | 0.28 | 24.56 | <0.0001 | H5N8 | Meat duck (6), breeder duck (1) |
|  | 13 | 2014/2/23–4/21 | 36.51N, 127.38E | 34.01 | 21 | 4.71 | 4.46 | <0.0001 | H5N8 | Layer (9), breeder duck (4), meat duck (3), broiler (1), goose (1)  Korean native chicken (1), mallard duck (1), mixed rearing (1) |
|  | 14 | 2015/2/2–2/15 | 36.99N, 127.31E | 10.25 | 7 | 0.39 | 17.86 | 0.0010 | H5N8 | Meat duck (4), Korean native chicken (2), breeder duck (1) |
|  | 15 | 2015/1/6–1/12 | 34.86N, 126.47E | 0.93 | 3 | 0.02 | 131.00 | 0.0122 | H5N8 | Meat duck (1), breeder duck (1), mallard duck (1) |
|  | 16 | 2015/1/24–1/30 | 35.25N, 127.42E | 0.39 | 5 | 0.20 | 24.56 | 0.0185 | H5N8 | Meat duck (5) |
|  | 17 | 2014/4/11–4/19 | 35.40N, 126.65E | 0.86 | 3 | 0.03 | 98.25 | 0.0377 | H5N8 | Meat duck (2), layer (1) |
| 6th | 1 | 2016/11/16–11/23 | 36.92N, 127.51E | 3.37 | 25 | 2.05 | 12.21 | <0.0001 | H5N6 | Meat duck (24), breeder duck (1) |
|  | 2 | 2017/2/27–4/4 | 36.26N, 127.09E | 28.44 | 14 | 1.14 | 12.32 | <0.0001 | H5N8 | Broiler breeder (6), Korean native chicken (2), layer (2), broiler (1),  Goose (1), white semi broiler (1), grandparent stock (1) |
|  | 3 | 2017/2/21–3/28 | 34.61N, 126.98E | 32.36 | 13 | 1.18 | 11.03 | <0.0001 | H5N8 | Meat duck (11), breeder duck (2) |
|  | 4 | 2017/6/6–6/12 | 35.91N, 127.09E | 35.13 | 15 | 1.70 | 8.80 | <0.0001 | H5N8 | Korean native chicken (13), duck (1), mixed rearing (1) |
|  | 5 | 2017/5/29–6/4 | 33.50N, 126.45E | 14.40 | 6 | 0.14 | 41.90 | <0.0001 | H5N8 | Korean native chicken and silkie (6) |
|  | 6 | 2016/12/16–2017/1/21 | 37.06N, 127.01E | 26.16 | 41 | 15.56 | 2.64 | <0.0001 | H5N6 | Layer (25), meat duck (6), laying breeder (2), broiler breeder (2),  Broiler (2), white semi broiler (2), Korean native chicken (2) |
|  | 7 | 2017/2/24–3/8 | 35.44N, 126.59E | 8.56 | 6 | 0.24 | 24.65 | 0.00056 | H5N8 | Meat duck (5), layer (1) |
|  | 8 | 2016/11/24–12/1 | 36.79N, 127.37E | 18.38 | 23 | 6.48 | 3.55 | 0.0010 | H5N6 | Meat duck (18), layer (2), breeder duck (2),  Korean native chicken (1) |
|  | 9 | 2017/1/22–1/28 | 37.91N, 127.27E | 12.85 | 3 | 0.02 | 139.67 | 0.0039 | H5N6 | Layer (3) |
|  | 10 | 2016/12/3–12/9 | 35.60N, 126.72E | 3.64 | 12 | 1.97 | 6.09 | 0.0042 | H5N6 | Meat duck (12) |
|  | 11 | 2017/5/30–6/5 | 35.60N, 129.10E | 32.73 | 5 | 0.20 | 24.94 | 0.0078 | H5N8 | Mixed rearing (3), Korean native chicken (2) |
|  | 12 | 2016/12/12–12/21 | 36.68N, 127.19E | 7.53 | 20 | 6.03 | 3.32 | 0.0306 | H5N6 | Layer (16), korean native chicken (3), meat duck (1) |
|  | 13 | 2016/12/7–12/14 | 37.11N, 127.50E | 17.52 | 28 | 10.56 | 2.65 | 0.0315 | H5N6 | Layer (17), meat duck (7), breeder duck (1), broiler breeder (1),  White semi broiler (1), Korean native chicken (1) |
| 10th | 1 | 2022/10/26–11/12 | 36.74N, 127.49E | 39.26 | 9 | 1.33 | 6.75 | <0.0001 | H5N1 | Meat duck (5), breeder duck (2), broiler (1), quail (1) |
|  | 2 | 2022/11/27–12/10 | 34.86N, 126.44E | 22.41 | 14 | 3.80 | 3.68 | 0.00031 | H5N1 | Meat duck (6), layer (5), breeder duck (2), broiler (1) |
| 12th | 1 | 2025/3/8–4/3 | 36.69N, 127.17E | 17.50 | 8 | 1.31 | 6.12 | <0.0001 | H5N1 | Layer (8) |

**Note:** In STPSS, a significant cluster represents a space-time scanning window with more observed cases than expected under the null hypothesis. Therefore, long-duration clusters may reflect prolonged or recurrent concentration within the same spatial window and do not necessarily indicate uninterrupted transmission continuity.
