# Supplementary material for: From Clustered to Sporadic: Structural Shifts in the Spatiotemporal Dynamics of HPAI Following the 2017 Policy Reinforcement in South Korea (2003–2025)
Source: Transbound Emerg Dis. 2026 Jul 7;2026:5747471. doi: 10.1155/tbed/5747471 (PMC13340132; doi:10.1155/tbed/5747471)
Supplement: Supplementary file 5 — Supporting Information 5 Figure S3. Heatmaps of spatiotemporal interaction intensity, D(s,t), derived from the space–time K‐function for major HPAI epidemic waves. These heatmaps illustrate the strength of local farm‐to‐farm transmission interaction within defined distance and time intervals. [file TBED-2026-5747471-s001.docx]

**Appendix Table 2.** Sensitivity analysis of KDE maxima across epidemic waves using Gaussian kernel bandwidths of 5 km, 10 km, and 15 km

| Wave | KDE max (5 km) | KDE max (10 km) | KDE max (15 km) |
| --- | --- | --- | --- |
| 1st | 1.990418 | 3.347561 | 3.700318 |
| 2nd | 4.819508 | 4.935354 | 4.675921 |
| 3rd | 25.773916 | 32.026066 | 34.540798 |
| 4th | 5.462434 | 9.580424 | 11.019073 |
| 5th | 53.967903 | 117.205132 | 121.687996 |
| 6th | 19.980698 | 35.106895 | 42.052635 |
| 7th | 1.854377 | 1.849416 | 2.301131 |
| 8th | 2.122962 | 3.690713 | 6.621974 |
| 9th | 3.144323 | 3.837258 | 3.859261 |
| 10th | 3.235588 | 4.884574 | 6.842293 |
| 11th | 5.396166 | 6.460357 | 6.605985 |
| 12th | 2.434417 | 4.868958 | 5.725768 |

**Note:** KDE maxima were recalculated under a consistent projected coordinate system (EPSG: 5179) with a raster cell size of 100 m. The 10 km bandwidth was used as the primary specification in the main analysis, while 5 km and 15 km bandwidths were used for sensitivity analysis.
